# The remarkable larval morphology of *Rhaebo nasicus* (Werner, 1903) (Amphibia: Anura: Bufonidae) with the erection of a new bufonid genus and insights into the evolution of suctorial tadpoles

**DOI:** 10.1186/s40851-024-00241-0

**Published:** 2024-09-30

**Authors:** Pedro Henrique dos Santos Dias, Jackson R. Phillips, Martín O. Pereyra, D. Bruce Means, Alexander Haas, Philippe J. R. Kok

**Affiliations:** 1 Leibniz Institut zur Analyse des Bioaffiliationersitätswandels, Zoologisches Museum Hamburg, Zentrum für Taxonomie und Morphologie, Martin-Luther-King-Platz 3, 20146 Hamburg, Germany; 2 Utah State University, 5305 Old Main Hill, Logan, Utah 84322 USA; 3 CONICET - Agencia INTA General Acha, Avellaneda 530 (8200), General Acha, La Pampa, Argentina; 4 Coastal Plains Institute and Land Conservancy, 1313 Milton Street, Tallahassee, Florida 32303 USA; 5 Department of Ecology and Vertebrate Zoology, Faculty of Biology and Environmental Protection, University of Łódź, 12/16 Banacha Str., Łódź, 90-237 Poland; 6 Life Sciences, The Natural History Museum, Cromwell Road, London, SW7 5BD UK; 7 Department of Biological Science, Florida State University, Tallahassee, Florida 32303 USA

**Keywords:** Evolution, Larval traits, Musculoskeletal system, Pantepui, Suctoriality, Systematics, Taxonomy

## Abstract

Tadpoles serve as crucial evidence for testing systematic and taxonomic hypotheses. Suctorial tadpoles collected in Guyana were initially assigned to *Rhaebo nasicus* through molecular phylogeny. Subsequent analysis of larval and adult morphological traits revealed synapomorphies within the clade encompassing *R. nasicus* and *R. ceratophrys*, prompting the recognition of a new genus described herein as *Adhaerobufo*. The new genus is distinguished from other bufonids by specific phenotypic traits including an enlarged, suctorial oral disc with distinct papillae arrangements, and the presence of certain muscles and narial vacuities at the larval stage. However, only a few adult external characteristics (e.g., enlarged eyelids, infraocular cream spot), seem to be reliably discriminative from related genera. This study underscores the significance of larval morphology in anuran systematics and offers new insights into the evolution of suctorial and gastromyzophorous larvae within bufonids.

**Supplementary Information:**

The online version contains supplementary material available at 10.1186/s40851-024-00241-0.

## Introduction

While adult traits have dominated the field of anuran systematics, biologists have long recognized the potential of larval morphology in better understanding evolutionary relationships. The earliest instance of a larval trait being used in this way can be traced to the late 19th century, when the French zoologist Fernand Lataste [1] proposed a new classification of frogs based on the position of spiracles. A few years earlier, Pizarro [2] had proposed the erection of the genus *Batrachychthis* for the bizarre tadpoles of *Pseudis*. During the following century, the impact of larval morphology on the systematics and taxonomy of anurans was further explored, especially by Noble, who published a series of papers [3–8] advocating the use of larval characters and natural history information in the classification of amphibians. Later, Orton [9] published a seminal paper in which she proposed that four major groups of frogs could be recognized based on larval characters (see also [10]).

Although some authors have argued against the usage of larval characters in taxonomic and systematic studies (e.g., [11, 12]), tadpoles are largely recognized as a source of useful evidence for such studies (e.g., [13–20]). For instance, Haas [21] used larval morphology to propose a new anuran phylogeny that anticipated several phylogenetic trends that have since been supported by the following generation of large-scale molecular studies (e.g., [22–23]).

The past two decades have witnessed constant growth in studies on tadpoles and the exploration of larval characters. Grosjean et al. [24] set a benchmark of the importance of larval characters in systematics, describing a new species based on its tadpole — *Clinotarsus penelope* (Ranidae). Several bizarre and previously unknown larval phenotypes have been described (e.g., [25–32]), and many new characters and synapomorphies for different groups have been proposed (e.g., [33–47]). In the present paper, we discuss the impact of larval morphology on the systematics and taxonomy of a clade of toads of the family Bufonidae.

The true toads, bufonids, are one of the most diverse and speciose anuran clades, with a nearly cosmopolitan distribution (found on all continents except Australia and Antarctica [48]). Currently, the 655 recognized species are allocated in 54 genera [48]. Bufonid diversity is also reflected in their numerous reproductive strategies and developmental modes (e.g., [49–58]). Bufonid tadpoles are also quite diverse, and while many genera have conserved a lentic-benthic larval phenotype (e.g., [59]), there is significant variation in ecology and morphology within the family, including *inter alia* suctorial (sucker mouth) and gastromyzophorous (belly sucker) forms, which represent adaptations to life in fast-flowing waters (e.g., [33, 60, 61]), phytotelma dwellers with endotrophic nutrition (e.g., [62–64]), open-water species with large, vascular crests [65], semiterrestrial tadpoles that live on wet rocks (e.g., [66]), and direct developers that retain larval traits (e.g., [55]). However, the tadpoles of many bufonid species remain unknown, and while some have assumed that their larval morphology will likely prove to be a typical benthic, lentic form, tadpoles continue to surprise us.

The Pantepui biogeographical region is located in northeastern South America, in the western Guiana Shield highlands, and is famed for its iconic table mountains of Proterozoic sandstone (locally known as “tepuis”). Tepuis are remnants of an enormous landmass (called the Roraima Supergroup or Mataui Formation) resulting from the sedimentation and subsequent uplifts of sandstones produced by the erosion of ancient Gondwanan highlands [67–69]. Over the last two decades a substantial number of new endemic amphibian species (e.g., [70–86], to only cite a few) and even endemic genera and families [87–89] have been described from the region, highlighting the importance of this often neglected biome in the evolution of Neotropical amphibians (see also [90, 91]).

During multiple expeditions in the Eastern Pantepui uplands and highlands of Guyana, DBM and PJRK observed and collected series of brightly colored tadpoles in fast-flowing mountain streams. Until recently, these larvae were assumed to be *Atelopus* cf. *hoogmoedi* based on overall external characteristics and microhabitat (fast-flowing mountain streams). However, a closer examination of the suctorial apparatus and recent molecular phylogenetic analyses indicated that these larvae do not belong to the genus *Atelopus* and should instead be assigned to *Rhaebo nasicus*. As the tadpole of *R. nasicus* is undescribed, we re-examined the larvae of these Pantepui “*Atelopus*” in detail. Our new findings strongly impact the understanding of the taxonomy of these toads and the evolution of bufonid tadpoles more generally.

## Materials and methods

### Sample determination, molecular data collection and analyses

#### Species assignment

Adults were assigned to *Rhaebo nasicus* based on external morphology characters, such as the eyelid projection. In Guyana, *R. nasicus* is the only species known to present this character-state. Additional to the phylogenetic placement, the tadpoles were assigned to the family Bufonidae based on the presence of larval synapomorphies of the family: anterolateral process of crista parotica absent, m. diaphragmatopraecordialis absent, lateral fibers of m. subarcualis rectus II–IV invading branchial septum, larval lungs rudimentary, and a single pair of infralabial papillae [21, 33]. In Guyana, there are four genera of bufonids: *Atelopus*, *Oreophrynella*, *Rhaebo*, and *Rhinella* [48]. All known tadpoles of *Atelopus* present a belly sucker [32], and *Oreophrynella* exhibits endotrophic development [53, 55, 92]. Tadpoles of *Rhaebo guttatus*, *Rhinella marina*, and *R. merianae* have been described [93]. Thus, these tadpoles could only be assigned to *R. nasicus*, *R. beebei*, *R. martyi*, or *R. nattereri* (the three latter being absent from our collection localities).

#### Tissue sampling, DNA extraction, amplification and sequencing

Genomic DNA was isolated from a small piece of the tail of a preserved tadpole (whole larva fixed in 99% ethanol in the field) from Mount Wokomung, Guyana (CPI10704; 05˚00’08”N, 59˚52’47”W at 1,573 m elevation) and from liver tissues of two adult *Rhaebo nasicus* (tissues fixed in 99% ethanol in the field) from two localities in Guyana: Kaieteur National Park (IRSNB14518 [PK1348]; 05˚08’N, 59˚25’W at ca. 540 m elevation), and the slopes of Maringma-tepui (PK1895; 05˚12’28”N, 60˚33’60”W at 1,060 m elevation).

Tissue samples were digested overnight at 56 °C in a solution of 5 µL of proteinase K and 100 µL of lysis buffer (100 mM NaCL, 100 mM Tris, 25 mM EDTA, 0.5% SDS). DNA extraction was performed using Sera-Mag™ SpeedBeads™ (Thermo Fisher Scientific) at a concentration of ca. 1.7 × (105 µl of digested tissue to 180 µL of beads) and eluted into 200 µl of 10 mM Tris buffer. Using polymerase chain reaction (PCR; for primers and PCR conditions see [94]), we amplified a fragment of the barcoding *16S ribosomal RNA gene* (*16 S*; 507 base pairs [bp]). PCR amplifications were confirmed on a 1% agarose gel, and negative controls were run on all amplifications to exclude contamination. PCR products were purified, and Sanger sequenced (along both strands using the same primers used for PCR) at the Natural History Museum’s (NHM, London, UK) sequencing facility. Chromatograms were assembled and edited in CodonCode Aligner 10.0.2 (Codon Code Cooperation, Dedham, USA). Novel sequences have been catalogued in GenBank (PQ200682–PQ200684). The newly generated sequences were uploaded onto BLAST NCBI (https://blast.ncbi.nlm.nih.gov/Blast.cgi) to identify the most similar sequences on GenBank.

#### Sequences editing and alignment settings

Based on the results of the BLAST analysis and guided primarily by the phylogenetic frameworks established by [95–97], we designed a sampling strategy to determine the placement of the sequenced specimens and elucidate their evolutionary relationships. Accordingly, our phylogenetic analyses focused on a mitochondrial fragment comprising the *12S RNA*, *tRNA valine*, and *16S RNA* genes (*12s-trna-val-16s*), complemented by three nuclear loci: a fragment of the *C-X-C motif chemokine receptor 4* gene (*cxcr4*), a fragment of the *proopiomelanocortin* gene (*pomc*), and a fragment of the *recombination activating 1* gene (*rag1*) for 82 bufonid specimens and 12 outgroups. Sequences were aligned using MAFFT v7 online software [98–99] with the strategy E-INS-i (for the *12s-trna-val-16s* fragment) and L-INS-i (for remaining fragments). Subsequently, the individual alignments were concatenated using SequenceMatrix v1.8 [100], resulting in a final alignment of 4,668 bp. The brachycephaloid *Ischnocnema guentheri* was used as the outgroup for tree rooting. Details regarding specimens, locality data, and GenBank accession numbers for the sequences used in our analyses are provided in Appendix MS1.

#### Maximum parsimony phylogenetic analysis

Phylogenetic analysis under Maximum Parsimony (MP) was performed in TNT version 1.6 [101, 102] using “New Technology” searches and treating gaps as a fifth state. The analysis utilized a combination of sectorial searches, ratchet, and tree-fusing techniques [103, 104] until the consensus tree was stabilized 10 times (see [103]). The parameters set of the search were: xmult = replications 10 ratchet 5 drift 5 fuse 5 consense 10. The support for each clade was evaluated by estimating two types of resampling support-measures for the datasets: (1) parsimony jackknife absolute frequencies (JAF; [105]) and (2) parsimony jackknife frequency differences (JGC; [106]). Jackknife supports were estimated performing 1000 replicates using “New Technology” searches with the following settings: xmult = hit 2 replications 12 xss fuse 3.

#### Maximum likelihood phylogenetic analysis

For Maximum Likelihood (ML) analysis, we initially determined the best partition scheme and corresponding models of nucleotide evolution using ModelFinder [107], as implemented in IQ-TREE 2.2.0 [108] with the command TESTNEWMERGEONLY. Coding genes were partitioned by codon position, while mitochondrial sequences (non-coding) were considered as a single partition. Defined initial partitions are detailed in Appendix MS2.

Subsequently, we searched for the best ML tree in IQ-TREE 2.2.0 with the partition scheme and models of nucleotide evolution selected by ModelFinder. We performed 10 independent searches with different values of perturbation parameter (-pers option) and the tree with the highest likelihood was selected as the optimal tree. For searches we consider edge linked-proportional partition model but separate substitution models and rate evolution between partitions (-spp option). The maximum-likelihood tree was conducted with 1000 ultrafast bootstrap (UFBoot) replicates (-B 1000 option; [109]).

#### Genetic distances

Uncorrected pairwise distances (UPDs) were calculated in PAUP* [110] for a dataset of the *16S* gene (507 bp, aligned in MAFFT under the 

G-INS-i strategy) and containing only sequences of species of *Rhaebo* (see Appendix MS3).

### Larval morphology

Larval morphology description is based on four tadpoles in developmental stages 25–38 (*sensu* [111]): three tadpoles (stages 25–26) housed in the National Museum of Natural History, Smithsonian Institution (USNM 592409-11) and one individual (CPI10704) at stage 38 (whose skeleton remains preserved [CPI10704]). All these larvae were originally collected as a single lot (CPI10704) in the Kamana Creek on Mount Kopinang of the Wokomung Massif in Guyana (site MK4; 05˚00′08′′N, 59˚52′47′′W at 1,573 m elevation). Terminology for external morphology characters follows [112, 113]. For the study of internal morphology, one tadpole in stage 38 (CPI10704) was submitted to the clearing and double staining protocol of [114]; the process was stopped after the alcian blue step, and the specimen was manually dissected for inspection of larval muscles. After photographic documentation of muscle characters, the palatoquadrate and the hyobranchial skeleton were gently disarticulated; upper and lower jaws were separated and the buccopharyngeal cavity exposed for study of its morphology. After recording characters from muscles and buccopharyngeal cavity, we concluded the clearing process for the study of the larval cranium and hyobranchial morphology. Terminology for the musculoskeletal system follows [47]; buccopharyngeal cavity follows Wassersug [19, 115].

Additionally, one tadpole in Gosner stage 25 (USNM 592409) was stained with phosphotungstic acid [116] and subjected to high-resolution micro-computed tomography (µCT). The tadpole was µCT-scanned using a Nikon X TH 225 ST 2x µCT scanner. Volumetric reconstruction was performed in Nikon CT agent and post-processed in VG Studio Max. Finally, we also examined other tadpoles of different bufonid species (See Appendix MS4).

### Adult morphology

We investigated the adult osteology of one individual of *Rhaebo nasicus* housed at the Royal Belgian Institute of Natural Sciences (IRSNB14518) using µCT scans. The individual was µCT-scanned using a YXLON FF20 CT. We also µCT-scanned two adult *R. ceratophrys* (UTA-A4061, UTA-A4062) and two adult *R. haematiticus* (UTA-A57567, UTA-A57572) housed in the herpetological collection of the University of Texas, Arlington, using a Nikon X TH 225 ST 2x µCT scanner. Some additional species were studied for osteology in (1) cleared and double stained specimens prepared following the techniques of Wassersug [117] and (2) reconstruction from µ-CT scans (see Appendix MS4).

### Evolution of suctoriality

We performed a parsimony optimization of tadpoles’ general ecomorphological types in the bufonid tree of life. The evolution of ecomorphological types was assessed using ancestral character state reconstruction as modeled on Fitch’s [118] optimization on the Portik et al.’s [97] topology using TNT [101, 102]. Ecomorphological information was taken from Vera Candioti et al. [57].

## Results

### Phylogenetic analyses and genetic distances

**Fig. 1:**
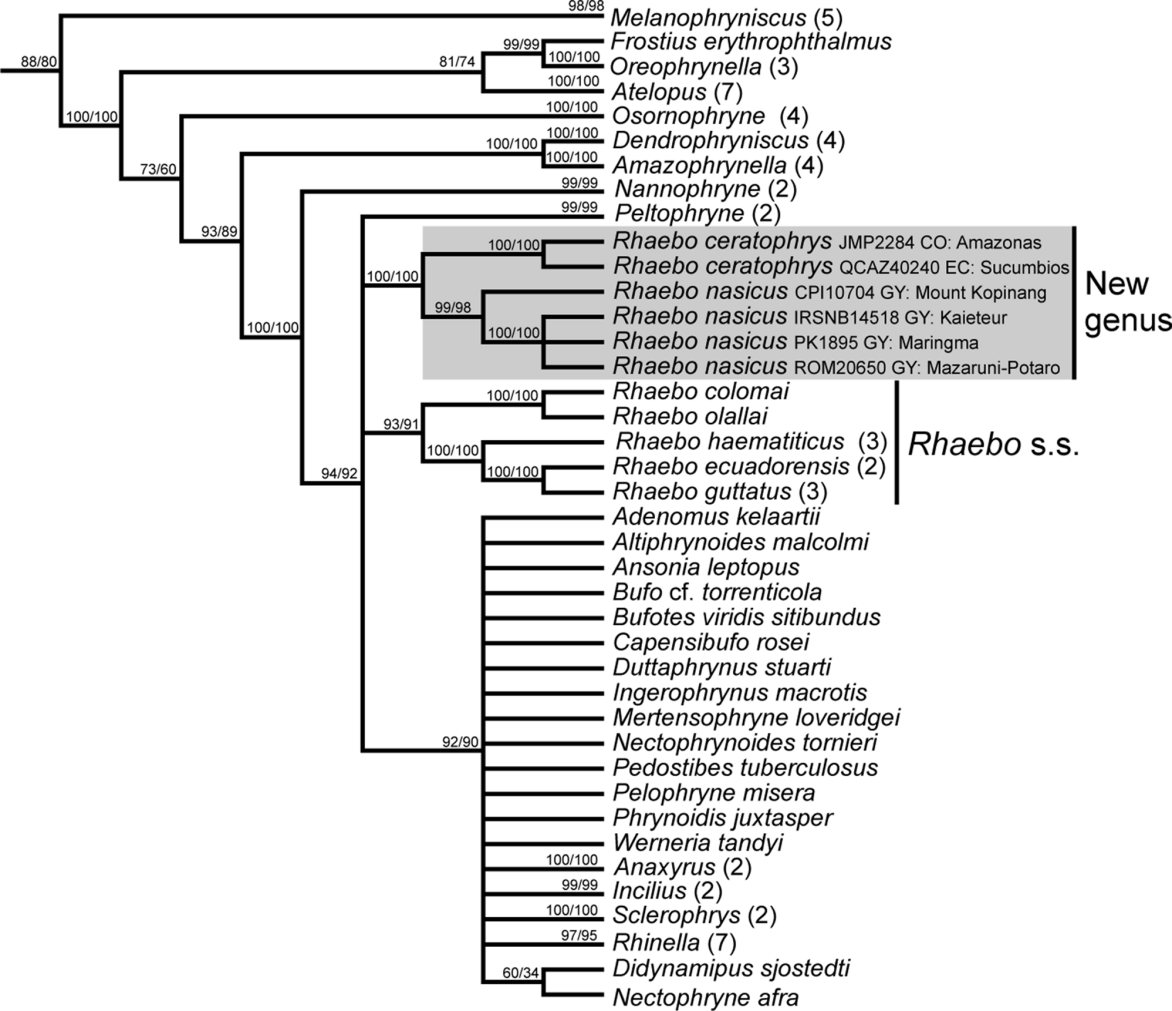
Summary tree of the maximum parsimony analysis depicting the relationships of *Rhaebo* and other Bufonidae. This tree represents the stabilized strict consensus derived from three most parsimonious trees (of length 15,782 steps). Values at nodes are parsimony jackknife frequencies (absolute/frequency differences). The numbers between parentheses following the names of genera denote the total condensed terminals at that tip. The complete MP strict consensus tree is shown in MS5

**Fig. 2:**
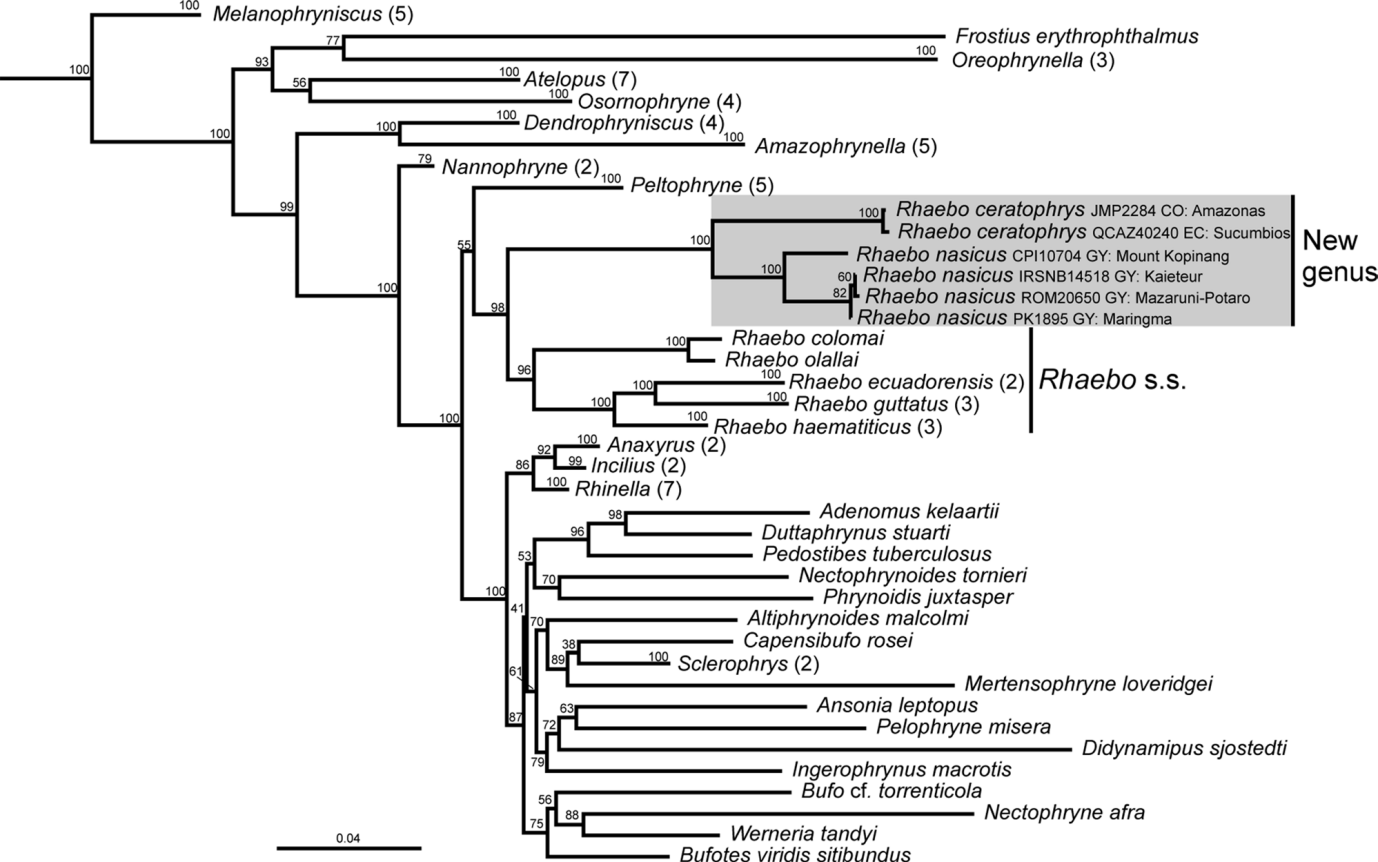
Summary tree of the maximum likelihood analysis depicting the relationships of *Rhaebo* and other Bufonidae. Values at nodes are bootstrap values. The numbers between parentheses following the names of genera denote the total condensed terminals at that tip. The complete ML tree is shown in MS6

A summary tree of *Rhaebo* and other bufonids is shown in Figs. 1 and 2 (for complete topologies, see MS5 and MS6). The topologies inferred by the MP and ML analyses consistently recover our new sequences within a highly supported clade along with *Rhaebo ceratophrys* and *R. nasicus* (JAF and JGC = 100%; UFBoot = 100%). The new sequences PQ200683 [IRSNB14518 (PK1348)] and PQ200684 (PK1895) were similar to the only available sequence of *Rhaebo nasicus* in GenBank (DQ158477 = ROM20650 [erroneously reported as ROM20560], from Tukeit in Kaieteur National Park, Guyana) with a genetic distance ranging from 0.21 to 0.43%. On the other hand, the sequence PQ200682 (CPI10704) was recovered as the sister lineage of that clade showing a genetic distance ranging from 4.84 to 5.78%. *Rhaebo ceratophrys* is, in turn, sister to the clade composed by the three new sequences and *R. nasicus* ROM20650. In the MP analysis (Fig. 1), the clade *R. ceratophrys* + *R. nasicus* collapses in a polytomy with (1) a moderately well supported clade (JAF = 93%, JGC = 90%) composed of the remaining included species of *Rhaebo*, (2) the highly supported *Peltophryne* (JAF and JGC = 100%) and (3) a moderately well supported clade (JAF = 92%, JGC = 89%) composed of the “New World” *Anaxyrus*, *Incilius* and *Rhinella*, and all the sampled “Old World” bufonids. In the ML analysis (Fig. 2), the internal topology of the clade *R. ceratophrys* + *R. nasicus* is mostly identical to the MP analysis, nevertheless, the relations of this clade with other bufonids are less conflicting. The clade *R. ceratophrys* + *R. nasicus* is recovered as sister of the remaining *Rhaebo* with high support (UFBoot = 98%), and *Rhaebo* is sister to *Peltophryne* with low support (UFBoot = 55%). Finally, *Rhaebo* + *Peltophryne* are sister to a well-supported clade (UFBoot = 100%) composed of the “New World” *Anaxyrus*, *Incilius*, and *Rhinella*, and all the sampled “Old World” bufonids.

### Larval morphology

#### External morphology (Figs. 3, 4 and 5)

**Fig. 3:**
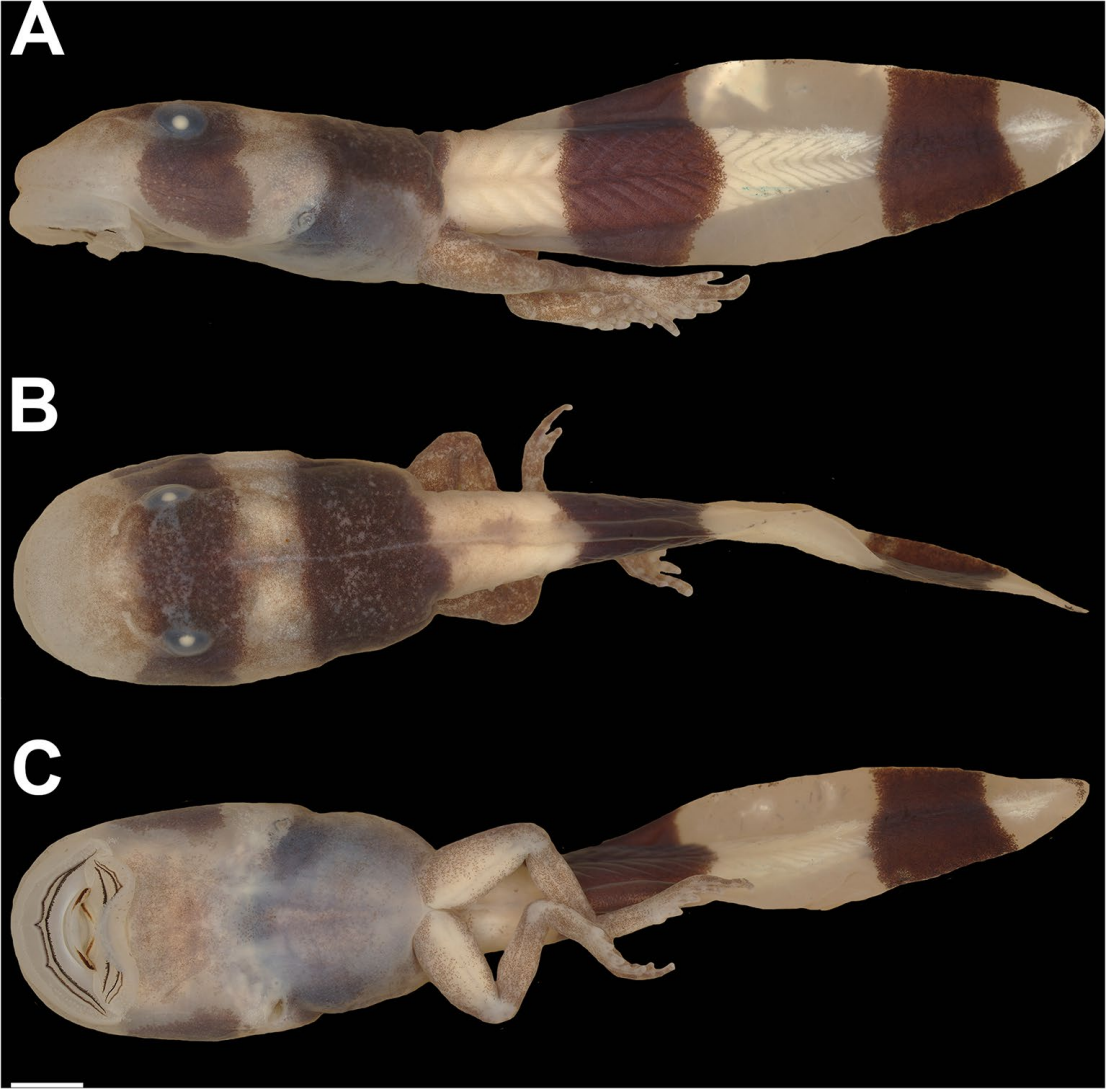
The tadpole of “*Rhaebo” nasicus* (CPI10704) at stage 38 in lateral (**A**), dorsal (**B**), and ventral (**C**) views. Scale bar = 1.0 mm. Photos by Pedro H. Dias

**Fig. 4:**
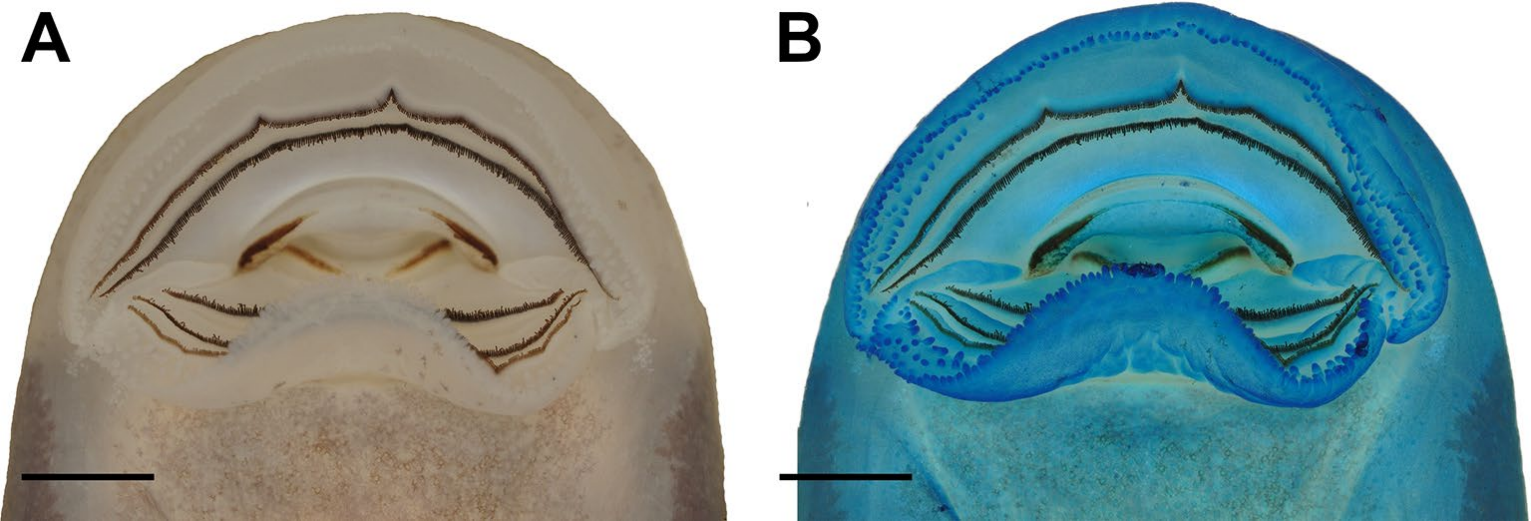
The oral disc of “*Rhaebo” nasicus* (CPI10704) tadpole at stage 38 in natural, preserved coloration (**A**) and stained with methylene blue to highlight anatomical features (**B**). Scale bars = 1.0 mm. Photos by Pedro H. Dias

Body compressed (Fig. 3A), elliptical in dorsal (Fig. 3B) and lateral views. Snout rounded in dorsal view, sloped in lateral view. Nostrils positioned dorsofrontally, elliptical, with a medial fleshy projection, anterolaterally directed. Eyes dorsal, laterally directed. Nasolacrimal duct visible (Fig. 3B). Spiracle sinistral, lateral, short, directed posteroventrally; centripetal wall presents as slight ridge. Digestive tract coiled; switchback point laterally dislocated from the center of abdominal region. Vent tube medial, directed posteroventrally, short, distal portion free from ventral fin. Tail higher than body; tail muscle almost reaching tail tip; tail tip rounded. Dorsal and ventral fins convex, about the same height; higher portions between the middle and posterior thirds of the tail. Dorsal fin originating on the tail. Lateral line system barely visible in preserved material. Oral disc (Fig. 4) enlarged, positioned and directed ventrally, laterally emarginate; a single, continuous row of conical, marginal papillae; no gaps in marginal papillation; submarginal papillae present, in all extension of the lower lip and laterally in the upper lip, with multiple parallel rows. Labial tooth row formula (LTRF) 2/3; A1 and A2 length subequal; P2 and P3 length subequal, slightly longer than P1. Jaw sheaths present, serrate, keratinized; upper jaw sheath arch-shaped (slightly less keratinized medially in the photographed specimen); lower jaw sheath V-shaped.

#### Color in life

**Fig. 5:**
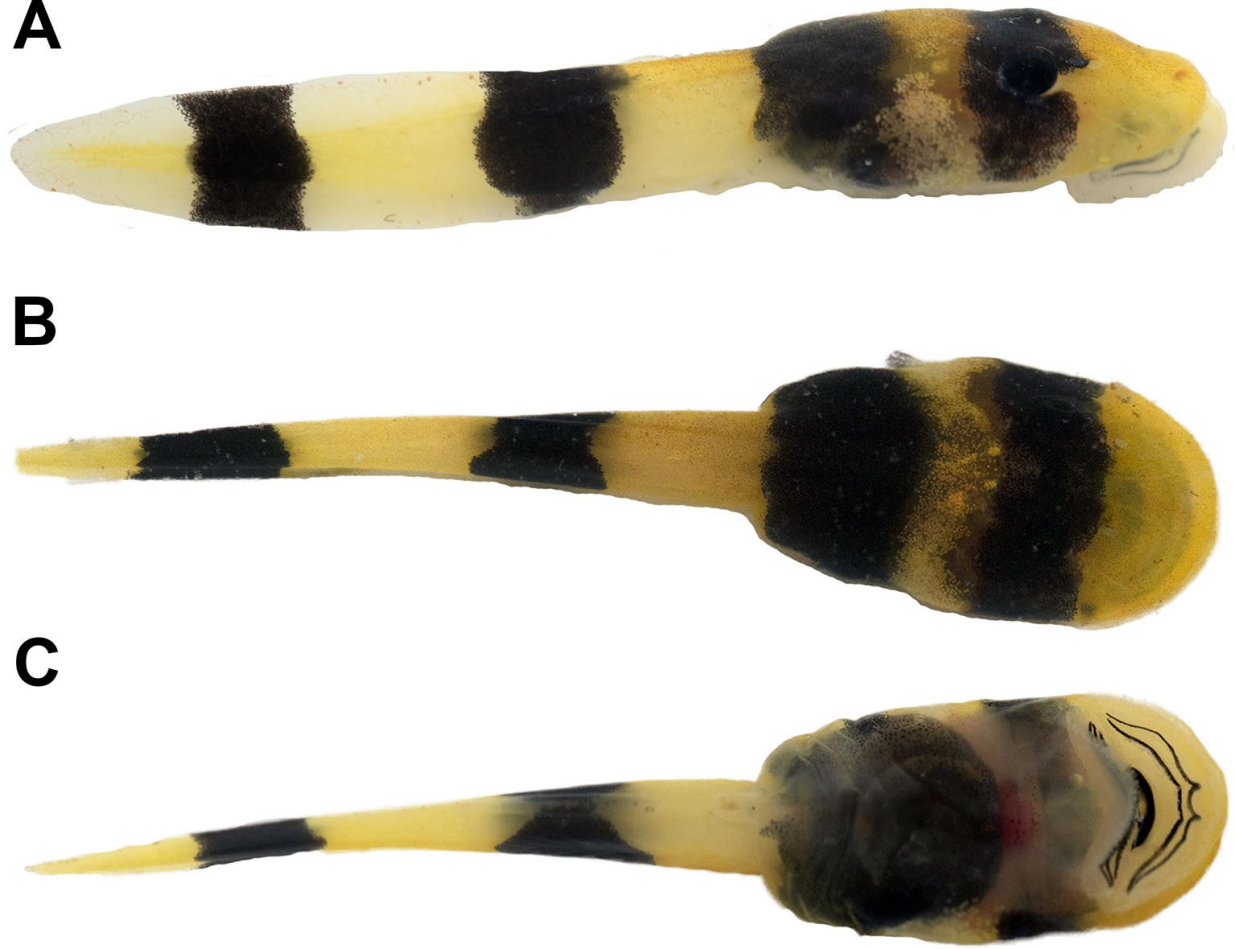
Living tadpole of “*Rhaebo” nasicus* in right lateral (**A**), dorsal (**B**), and ventral (**C**) views. Photos by D. Bruce Means

In life (Fig. 5), the overall coloration is yellow-gold dorsally and ventrally but is divided into five yellow-gold bands by four transverse dark bands of approximately the same width. The tadpole snout is yellow-gold from the tip to the eye, then the narrowest yellow-gold band encircles the midbody with an overwash of dark pigment. The posterior one-third of the tadpole body is densely black set off by the first of three yellow bands on the tail, the tip of which is the last yellow-gold band. The oral disc is translucent. Ventral views reveal a fading of the dark banding pattern along the body, with translucent skin offering glimpses of internal organs. Upon preservation, the vibrant hues subside, and the yellow-gold bands take on a cream-colored appearance separated by dark brown bands with scattered light brown blotches (Fig. 2).

#### Buccopharyngeal cavity (Fig. 6)

**Fig. 6:**
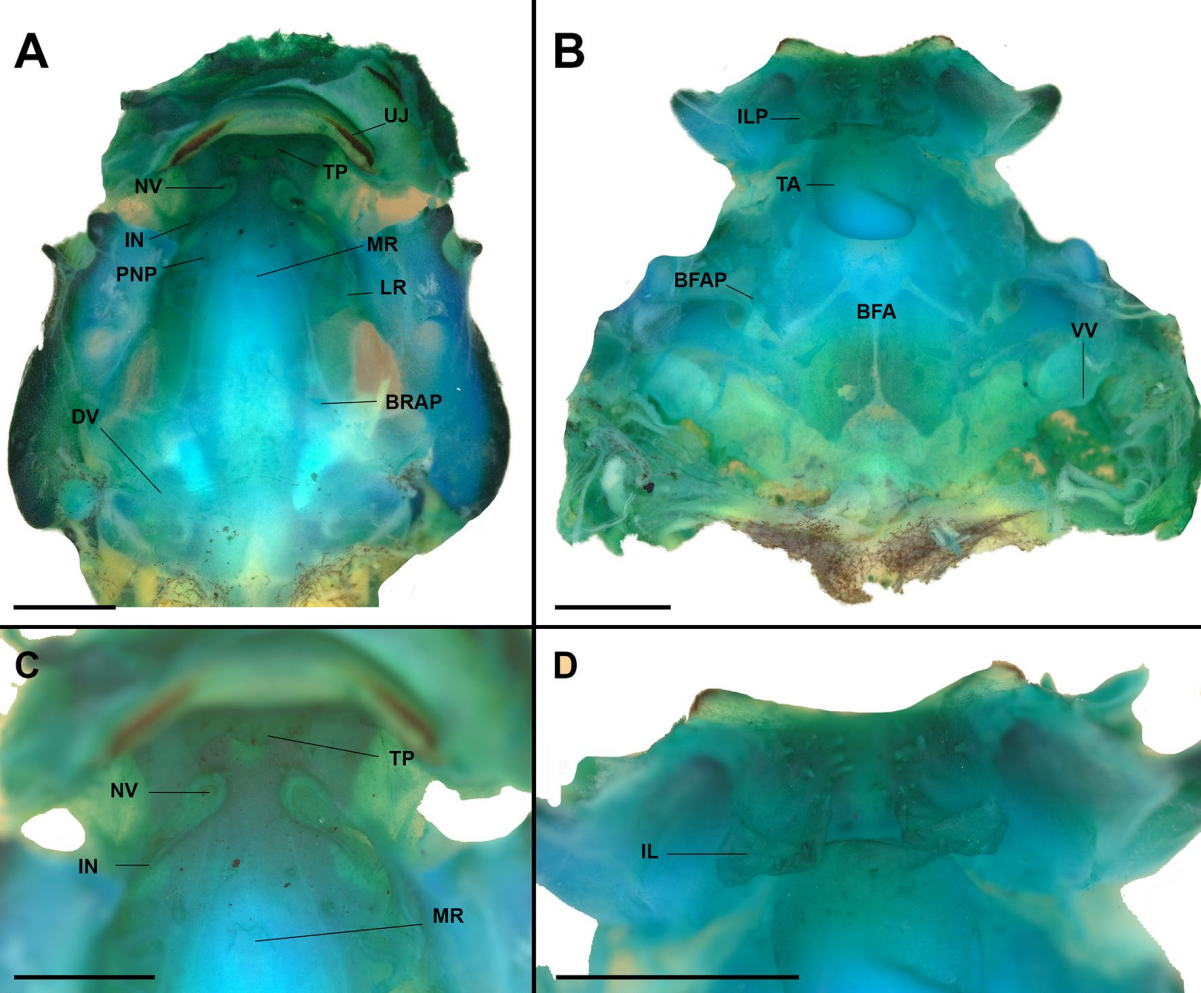
The buccopharyngeal cavity of “*Rhaebo” nasicus* (CPI10704) tadpole at stage 38. Buccal roof (**A**) and floor (**B**) morphologies, with details of the pre- and postnarial arenas (**C**) and of the infralabial and lingual (**D**) regions. BFA, buccal floor arena; BFAP, buccal floor arena papillae; BRAP, buccal roof arena papillae; DV, dorsal velum; ILP, infralabial papillae; IN, internal nares; LR, lateral ridge; MR, median ridge; NV, narial vacuities; PNP, postnarial arena papillae; TA, tongue anlage; TP, triangular projection; UJ, upper jaw sheath; VV, ventral velum. Scale bars = 1.0 mm. Photos by Pedro H. Dias

Buccal roof (Fig. 6A) triangular. Prenarial arena (Fig. 6C) rectangular, with a triangular protuberance. Internal nares elliptical (Fig. 6C), transversally oriented; posterior valve free, with small, triangular projections in the anterior wall. Vacuities present, circumscribed by margins of inner nares. Postnarial arena diamond-shaped, two conical, short postnarial papillae. Lateral ridge papillae short, trifurcated. Median ridge low, triangular, with a medial notch at its apex. Buccal roof arena poorly delimited, defined by a single pair of conical papillae each side. Glandular zone poorly defined. Dorsal velum medially continuous, devoid of papillae or projections, arch shaped.

Buccal floor (Fig. 6B) triangular. Single pair of flat, wide, branched, infralabial papillae; small papilla-like structures after mouth opening (Fig. 6D). Lingual bud well developed, rounded; lingual papillae absent. Buccal floor arena bell-shaped; 7–8 papillae each side. Buccal floor arena lacking pustulations. Prepocket papillae and pustulation absent. Buccal pockets deep, wide, oblique slit shaped. Ventral velum present; spicular support conspicuous; medial notch absent; secretory pits poorly developed; secretory ridges present. Branchial basket triangular, short, poorly developed, wider than long.

#### Larval cranium (Fig. 7)

**Fig. 7:**
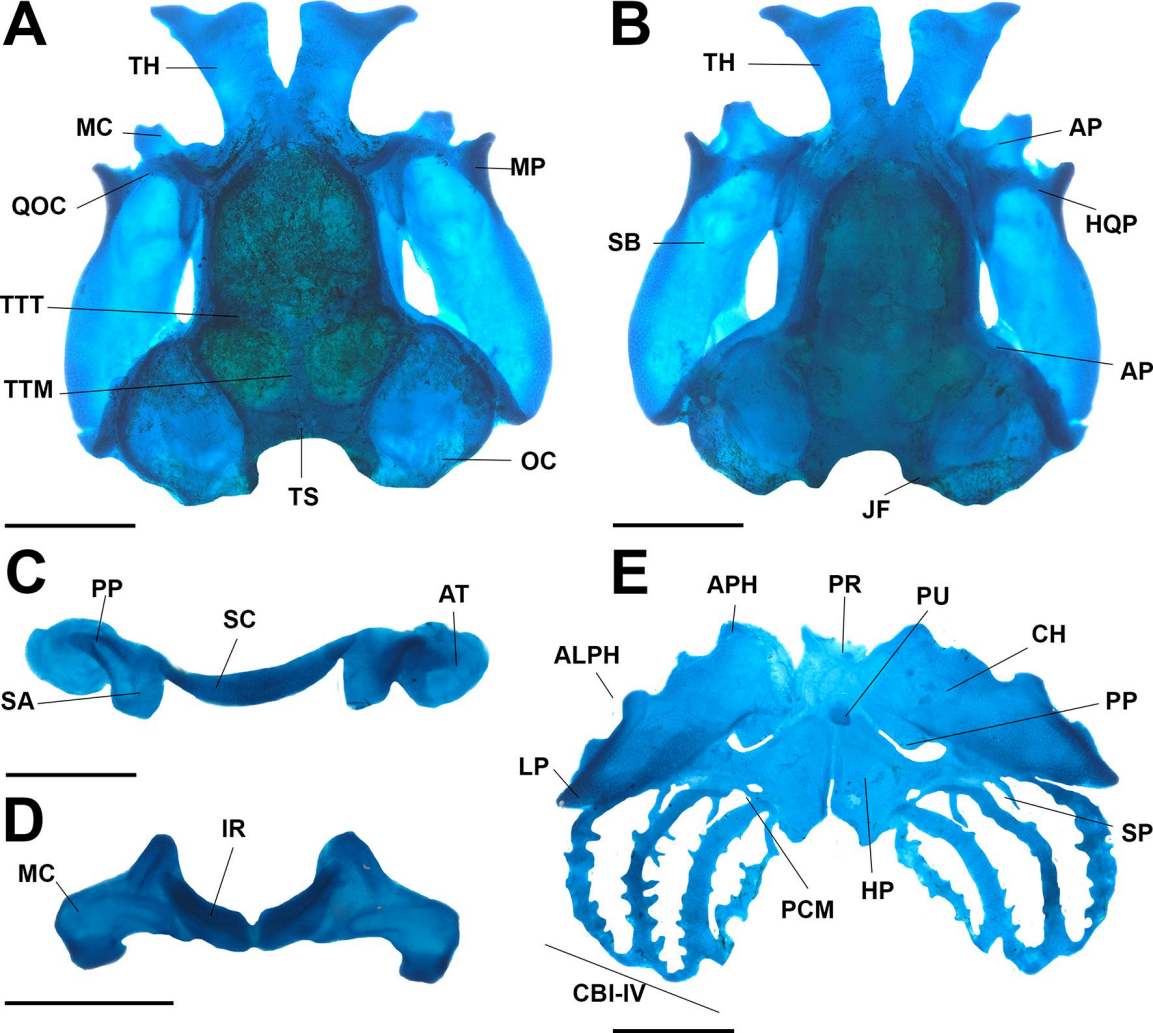
The larval cranium of “*Rhaebo” nasicus* (CPI10704) tadpole at stage 38. Dorsal (**A**), ventral(**B**) views, details of the suprarostral (**C**) and Meckel’s cartilage (**D**), and hyobranchial apparatus (**E**). ALPH, antelateral process hyalis; AP, articular process; APH, anterior process hyalis; AT, adrostral tissue; CB, constrictor branchialis; CH, ceratohyal; HP, hypobranchial plate; HQP, hyoquadrate process; IR, infrarostral cartilage; JF, jugular foramen; LP, lateral process; MC, Meckel`s cartilage; MP, muscular process; OC, otic capsule; PCM, proximal commissure; PP, posterior process; PU, process urobranchialis; QOC, quadro-orbital commissure; SA, suprarostral ala; SB, subocular bar; SC, suprarostral copora; SP, spicule; TH, trabecular horns; TS, tectum synoticum; TTM, taenia tecti medialis; TTT, taenia tecti transversalis. Scale bars = 1.0 mm. Photos by: Pedro H. Dias

Neurocranium longer than wide; greatest width at the subocular bar level (Fig. 7A–B). Suprarostral cartilage (Fig. 7C) formed by the suprarostral alae and suprarostral corpora; both corpora are medially fused and connected to the proximal region of the triangular alae. An adrostral tissue mass is present close to the posterior process of the alae (Fig. 7C); under dissection, it did not appear to be chondrified, but histological analysis should be done to confirm. Ethmoidal region short; trabecular horns long, diverging in a “V” pattern; trabecular horns greatly expanded anteriorly. Basicranial fenestra weakly chondrified, partially occluded by a thin membrane. Taenia tecti medialis and transversalis present and confluent (Fig. 7A), dividing the frontoparietal fontanelle in three. Orbital cartilage low. Otic capsules robust, rhomboidal in dorsal view, representing ca. 1/4 of chondrocranium length; synotic tectum connects the two capsules. Palatoquadrate, thin in lateral view, attached to neurocranium through a wide anterior quadratocranial commissure and an almost perpendicular ascending process. Articular process wide. Muscular process triangular, well-developed, and curved dorsomedially. Connection between the tip of the muscular process and the neurocranium through a chondrified quadrato-orbtial commissure. Palatoquadrate C-shaped, clearly concave; posterior curvature of palatoquadrate reaching the level of the otic capsules.

In the lower jaw (Fig. 7D), Meckel’s cartilage sigmoid, transversely oriented, almost perpendicular to the chondrocranium longitudinal axis. Infrarostral cartilages rectangular in frontal view, curved, joined at the symphysis (Fig. 7D).

Ceratohyals (Fig. 7E) long, flat, and subtriangular; anterior margin with well-developed anterior and anterolateral processes; posterior processes triangular and long. Ceratohyals confluently joined by a chondrified pars reuniens. Basibranchial rectangular, with rounded urobranchial process present. Basihyal absent. Hypobranchial plates long, triangular. Branchial basket with four curved ceratobranchials bearing lateral projections. Ceratobranchial I with a triangular anterior branchial process, continuous with the hypobranchial plate. Ceratobranchials II and III joined by the proximal commissure. Four long, curved spicules projecting dorsally from the ceratobranchials. Ceratobranchials distally joined by terminal commissures.

#### Muscles (Figs. 8, 9 and 10)

**Fig. 8:**
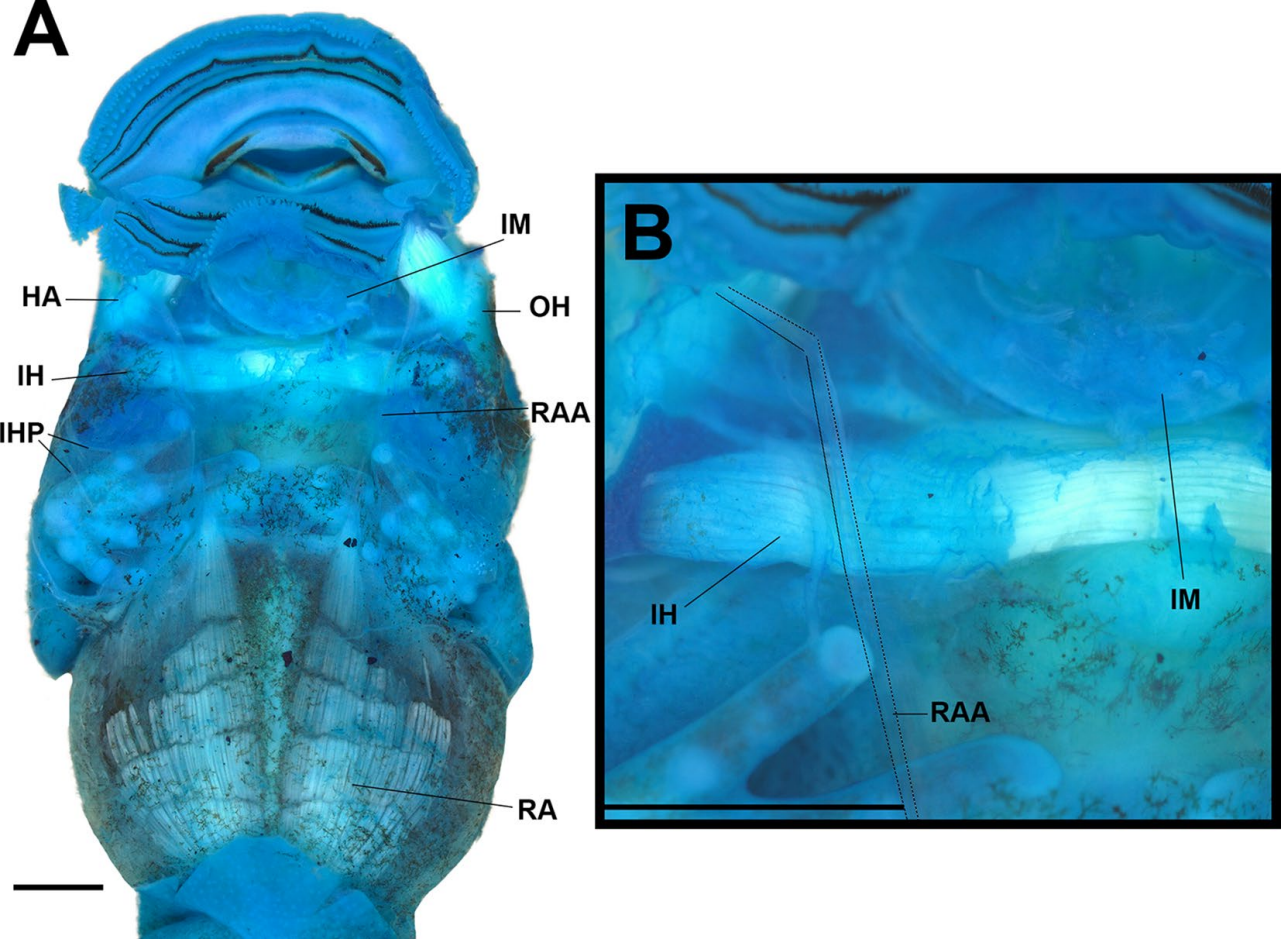
The larval muscles of “*Rhaebo” nasicus* (CPI10704) tadpole at stage 38 in ventral view (**A**); detail of the tendon of the m. rectus abdominis anterior (**B**). HA, hyoangularis; IH, interhyoideus; IHP, interhyoideus posterior; IM, intermandibularis; OH, orbitohyoideus; RA, rectus abdominis; RAA, rectus abdominis anterior. Scale bars = 1.0 mm. Photos by: Pedro H. Dias

**Fig. 9:**
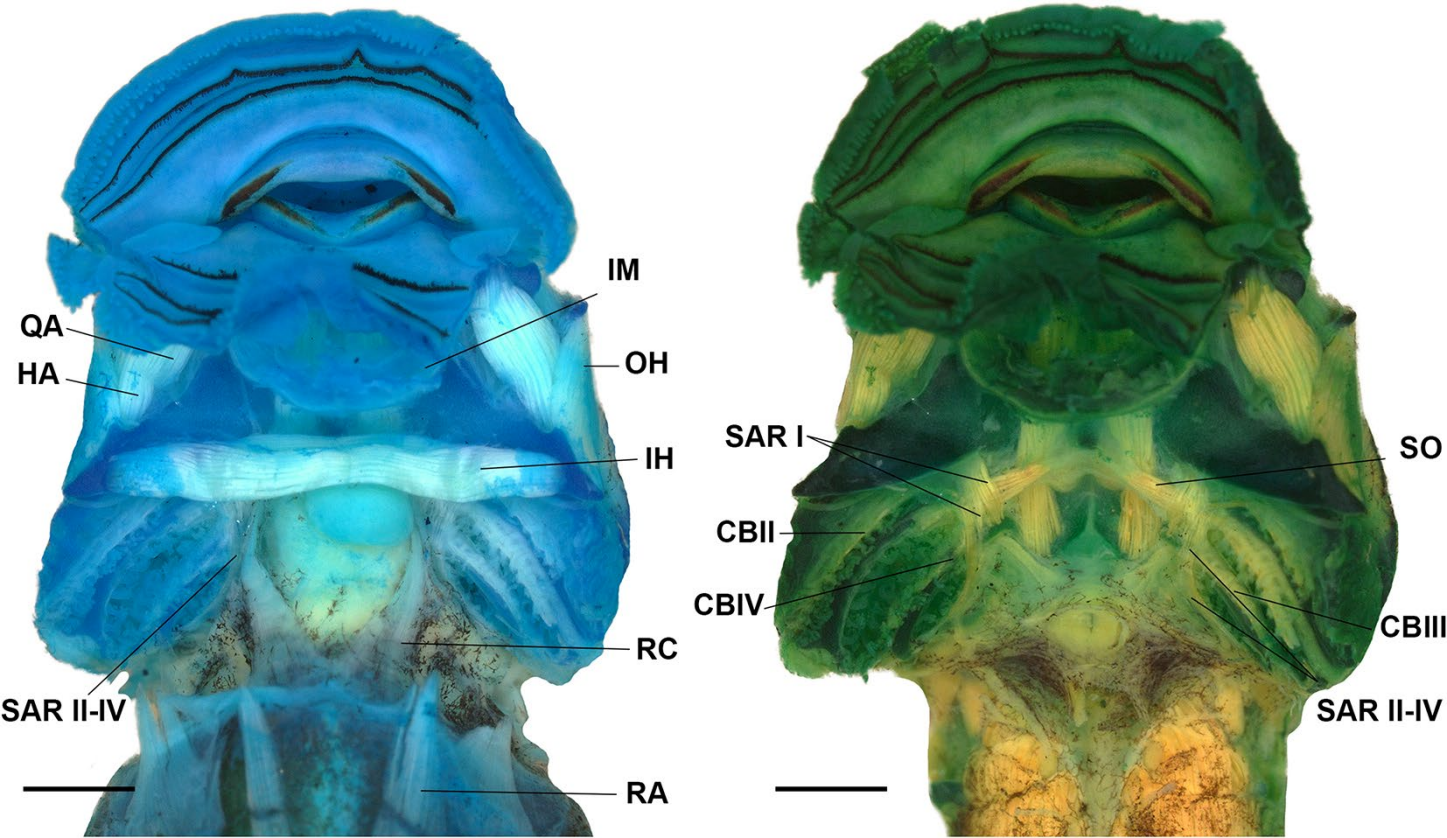
The larval muscles of “*Rhaebo” nasicus* (CPI10704) tadpole at stage 38 in ventral view. CB, constrictor branchialis; HA, hyoangularis; IH, interhyoideus; IM, intermandibularis; OH, orbitohyoideus; QA, quadrato-angularis; RA, rectus abdominis; RC, rectus cervicis; SAR I, subarcualis rectus I; SAR II–IV, subarcualis rectus II–IV; SO, subarcualis obliquus. Scale bars = 1.0 mm. Photos by Pedro H. Dias

**Fig. 10:**
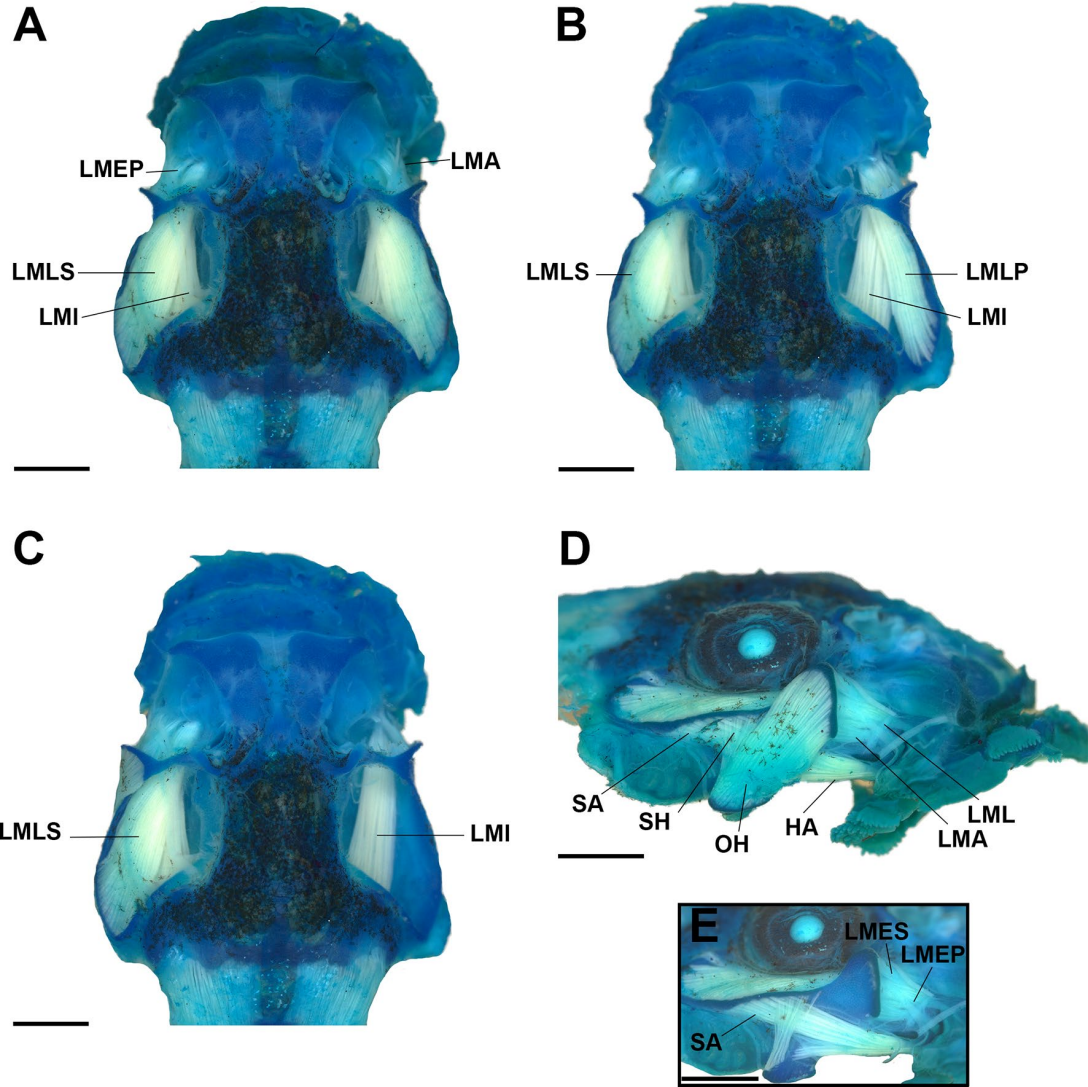
The larval muscles of “*Rhaebo” nasicus* (CPI10704) tadpole at stage 38 in dorsal (**A-C**) and lateral (**D-E**) views. LMA, levator mandibulae articularis; LMEP, levator mandibulae externus profundus; LMES, levator mandibulae externus superficialis; LMI, levator mandibulae internus; LMLS, levator mandibulae longus superficialis; OH, orbitohyoideus; SA, suspensorioangularis; SH, suspensoriohyoideus. Scale bars = 1.0 mm. Photos by: Pedro H. Dias

We identified 32 muscles (Table 1); most of *Rhaebo nasicus* muscles followed general patterns of origin and insertion of other bufonids and other anurans (Figs. 8, 9 and 10). Interestingly, the lateral fibers of the subarcualis rectus II–IV invade the interbranchial septum IV (Fig. 9) and the presence of the rectus abdominis anterior.

**Table 1:** Muscles origin and insertion in the larva of “*Rhaebo*” *nasicus*

Muscle	Origin	Insertion	Comments
**Mandibular group,*n***. **trigeminus (c.*n*. V) innerved**			
Levator mandibulae longus superficialis	External posterior margin of subocular bar	Dorsomedial Meckel’s cartilage	Via long tendon
Levator mandibulae longus profundus	External margin (curvature) of subocular bar	External margin of suprarostral ala	Via a long tendon
Levator mandibulae longus internus	Ventral otic capsule and processus ascendens	Lateral Meckel`s cartilage	Via a long tendon
Levator mandibulae externus superficialis	Inner muscular process (superior)	Adrostral tissue mass	
Levator mandibulae externus profundus	Inner muscular process (medial)	Distal suprarostral ala	Share a tendon with LMLP
Levator mandibulae articularis	Inner muscular process (inferior)	Dorsal Meckel’s cartilage	
Levator mandibulae lateralis	Articular process	Adrostral tissue mass	
Submentalis (intermandibularis anterior)	-	-	
Intermandibularis	Median aponeurosis	Ventromedial Meckel’s cartilage	
Mandibulolabialis	Ventromedial Meckel’s cartilage	Lower lip	
Mandibulolabialis superior	-	-	
**Hyoid group**,** n. facialis (c.n. VII)**			
Hyoangularis	Dorsal ceratohyal	Retroarticular process of Meckel’s cartilage	
Quadratoangularis	Ventral palatoquadrate	Retroarticular process of Meckel’s cartilage	
Suspensorioangularis	Ventral palatoquadrate	Retroarticular process of Meckel’s cartilage	
Orbitohyoideus	Muscular process	Lateral edge of ceratohyal	
Suspensoriohyoideus	Posterior descending margin of muscular process and subocular bar	Lateral process of ceratohyal	
Interhyoideus	Median aponeurosis	Ventral ceratohyal	
**Branchial group**,** n. Glossopharyngeus (c.n. IX) and vagus (c.n. X)**			
Levator arcuum branchialium I	Lateral subocular bar	Ceratobranchial I	
Levator arcuum branchialium II	Lateral otic capsule	Ceratobranchial II	
Levator arcuum branchialium III	Lateral otic capsule	Ceratobranchial III	
Levator arcuum branchialium IV	Lateroventral otic capsule	Ceratobranchial IV	
Tympanopharyngeus	Lateroventral otic capsule	Ceratobranchial IV	
Constrictor branchialis I	-	-	
Constrictor branchialis II	Branchial process II	Terminal commissure I	
Constrictor branchialis III	Branchial process II	Terminal commissure II	
Constrictor branchialis IV	Ceratobranchial III	Terminal commissure II	I
Subarcualis rectus I	Posterior lateral base of ceratohyal	Branchial processes II and III, and ceratobranchial I	
Subarcualis rectus II-IV	Ceratobranchial IV	Ceratobranchial II	Lateral fibers invading the interbranchial septum IV
Subarcualis obliquus II	Urobranchial process	Ceratobranchials II	Single slip
Diaphragmatobranchialis	Peritoneum (diaphragm)	Distal Ceratobranchial III	
**Spinal group**,** spinal nerve innervation**			
Geniohyoideus	Hypobranchial plate	Infrarostral cartilage	At the level of CB III
Rectus abdominis	Peritoneum (diaphragm)	Pelvic girdle	Six open myomers
Rectus abdominis anterior	Peritoneum (diaphragm)	Ventral palatoquadrate	Very short fibers; via a long tendon
Rectus cervicis	Peritoneum (diaphragm)	Branchial process III	

#### Visceral components

Digestive tract short; coiled gut with switchback point sinistral. Liver enlarged, occupying a significant portion of the abdominal cavity. Lungs short, inflated, pigmented.

### Adult morphology

The adult morphology of both *Rhaebo nasicus* and *R. ceratophrys* has been widely reviewed in the literature, including aspects of their osteology (e.g., [96, 119–121]). The most obvious shared character between *R. nasicus* and *R. ceratophrys* is the presence of an enlarged eyelid in both species (although more distinctly projecting in *R. ceratophrys*). An infraocular cream spot is also evident in adult specimens of both species. Additionally, both species share a narrow sphenethmoid (see below).

Pramuk defined the “*Bufo guttatus* group” (= *Rhaebo*) as presenting two unique, unreversed synapomorphies: the sphenethmoid in ventral view is distinctively broad, and the posterior process of the prootic is prominent and notched ([121]:434). Pramuk did not consider *B. nasicus* to be part of that clade and stressed that *B. nasicus* and the *B. guttatus* group share the presence of a well-developed omosternum and an elongated transverse process of vertebra VI ([121]:434). However, most of the osteological characters for *B. nasicus* were missing in her analysis.

**Fig. 11:**
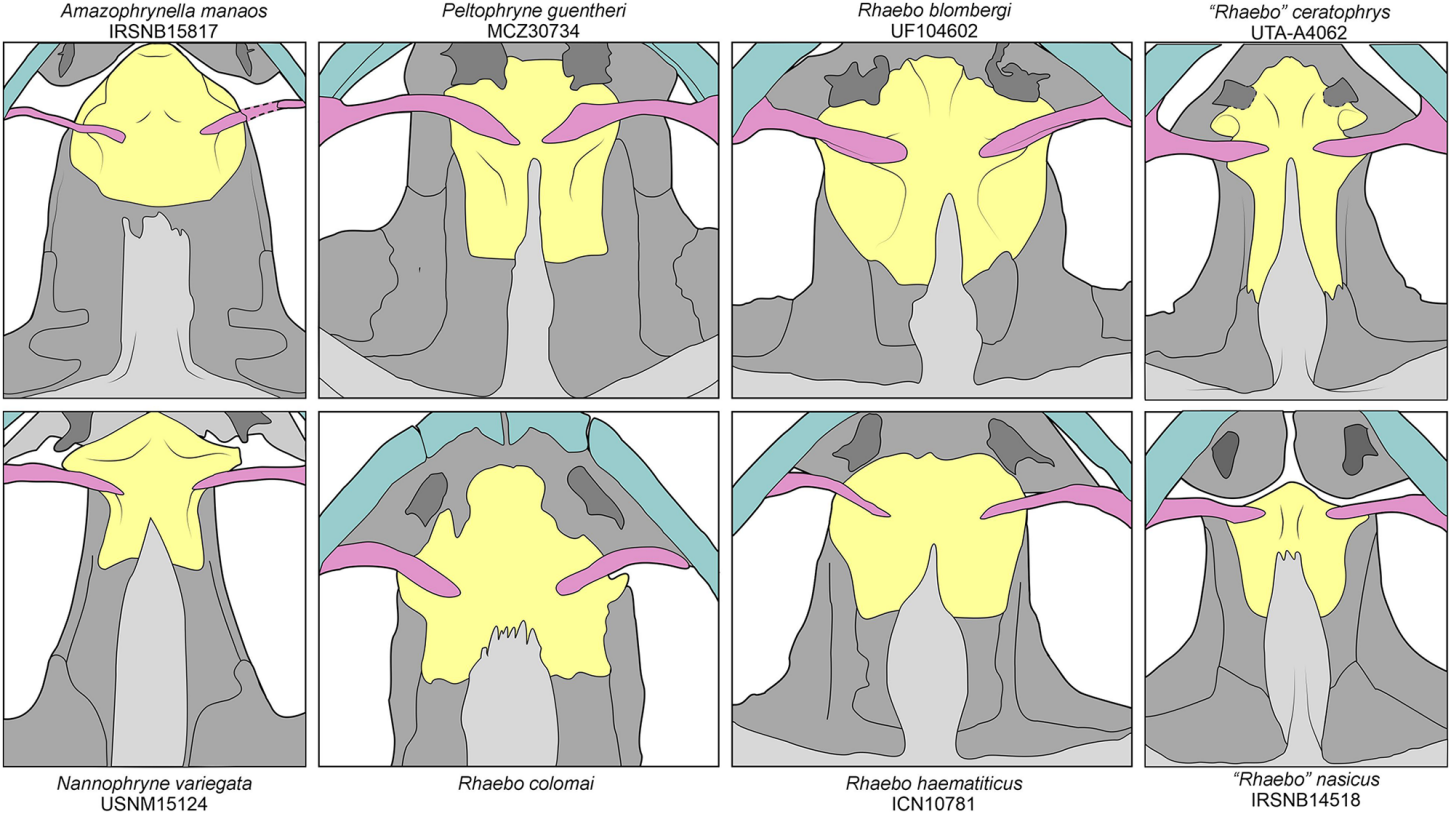
Variation in the sphenethmoid morphology in *Amazophrynella*, *Nannophryne*, *Peltophryne*, and *Rhaebo* sensu lato (sl). Different cranial bones are colored as follows for reference: blue (maxilla and premaxilla), dark grey (vomers), light grey (parasphenoid), pink (palatines), and yellow (sphenethmoid). Figures of *Peltophryne guentheri* and *Rhaebo colomai* were redrawn and slightly modified from [95] and [121]

Ron et al. ([95]:354) proposed a redefinition for the states “narrow” and “distinctively broad” for the sphenethmoid condition of Pramuk ([121]: ch35), considering the species of *Rhaebo* to have a “wide condition” due to the lateral edges of the sphenethmoid being in contact with the frontoparietals. In species where the frontoparietals do not extend to the anterior portion of the orbit (e.g., *Peltophryne*), a more accurate definition of the “wide” condition of the sphenethmoid could be as follows: the sphenethmoid reaches the margin of the orbit immediately posterior to the palatines. Both “*R.*” *ceratophrys* and “*R.*” *nasicus* have a narrow condition of the sphenethmoid (i.e, the sphenethmoid does not reach the margin of the orbit immediately posterior to the palatines), differentiating them from other *Rhaebo* (Fig. 11). The narrow condition of the sphenethmoid is also observed in bufonids closely related to “*R.*” *ceratophrys* and “*R.*” *nasicus*, such as *Amazophrynella*, *Nannophryne* and *Peltophryne*, which suggests the wide sphenethmoid to be a synapomorphy of *Rhaebo *sensu stricto.

Regarding the second synapomorphy of *Rhaebo* proposed by Pramuk ([121]; i.e, posterior process of the prootic prominent and notched), Ron et al. [95] pointed out a perceived error in the identification of the anterior prootic processes (sic) by Pramuk [121], stating that they were, in fact, the occipital condyles, which are part of the exoccipital rather than the prootic. However, both structures are clearly illustrated and identified in Fig. 5A of Pramuk’s work [121], suggesting a possible misunderstanding of these structures by Ron et al. [95], so we follow Pramuk [121]. In this regard, “*R.*” *ceratophrys* and “*R.*” *nasicus* have prominent and notched posterior process of the prootic as other species of *Rhaebo*, but also seen in some species of *Peltophryne* ([121]: Fig. 2).

### Comments on the taxonomic and systematic history of “*Rhaebo” ceratophrys* and “*Rhaebo” nasicus*

*Rhaebo ceratophrys* was first described in 1882 by Boulenger ([122] as *Bufo ceratophrys*) based on a juvenile specimen from Ecuador (BMNH 1880.12.5.151). The species was characterized by a unique feature, a long eyelid projection. Since then, the species has been transferred to several different species groups within the former genus *Bufo*. For instance, Gallardo [123] allocated it in the *B. marinus* group, whereas Cei [124], Hoogmoed [120] and Pramuk [121] considered the species as belonging to the *B. typhonius/margaritifer* group.

The taxonomic history of *Rhaebo nasicus* has also been convoluted. Werner [125] found a specimen of an unknown toad (IRSNB1015, formerly IRSNB4792), which he named *Bufo nasicus*. Later, studies of the contents of its digestive tract suggested a South American origin (Smith and Laurent 1950), and eventually Hoogmoed [120] accessed additional specimens from Guyana and Venezuela. He compared these individuals with the redescription and illustrations made by Smith and Laurent [126], identifying them as the *Bufo nasicus* of Werner [125]. Hoogmoed [120] redescribed the species and suggested it to be restricted to the Guiana Shield. Hoogmoed [120] noted the presence of an enlarged eyelid in *B. nasicus* and argued that the shared presence of such an eyelid in *B. nasicus* and in *B. ceratophrys*, as well as similar color patterns indicate a close relationship between the two species. Hoogmoed [120] also noted that *B. ceratophrys* was much smaller than *B. nasicus* — a misinterpretation repeated by others (e.g., [127]), since the holotype of *B. ceratophrys* is a juvenile.

Pramuk [121] performed an extensive phylogenetic analysis of Bufonidae, using both morphology and molecular data. She recovered *Bufo nasicus* as sister to other species of the *Bufo guttatus* group of Blair [128] in all analyses (morphological, mitochondrial genes, nuclear genes, and combined analyses). She also examined specimens of *B. ceratophrys* (her appendix 1; p.443), but the species does not appear in any of her phylogenetic hypotheses. Concurrently, Frost et al. [23] resurrected the genus *Rhaebo* to accommodate species of the *Bufo guttatus* group of Blair [128]. They also transferred *Bufo ceratophrys* and *Bufo nasicus* to the genus *Rhinella*. Finally, Frost [23], considering the evidence of Pramuk [121], transferred *Rhinella nasica* to *Rhaebo*, which remains the current taxonomy.

Fenolio et al. [119] recognized that the diagnosis of *Rhaebo ceratophrys* (as *Rhinella ceratophrys*) proposed by Hoogmoed [120] needed to be revised in the light of new collections. They performed a detailed morphological study and provided a new diagnosis for the species, recognizing it as having: (1) triangular projecting dermal flaps on the eyelids, (2) projecting dermal flaps at the corners of mouth, and (3) a larger adult size ([119]:10).

**Fig. 12:**
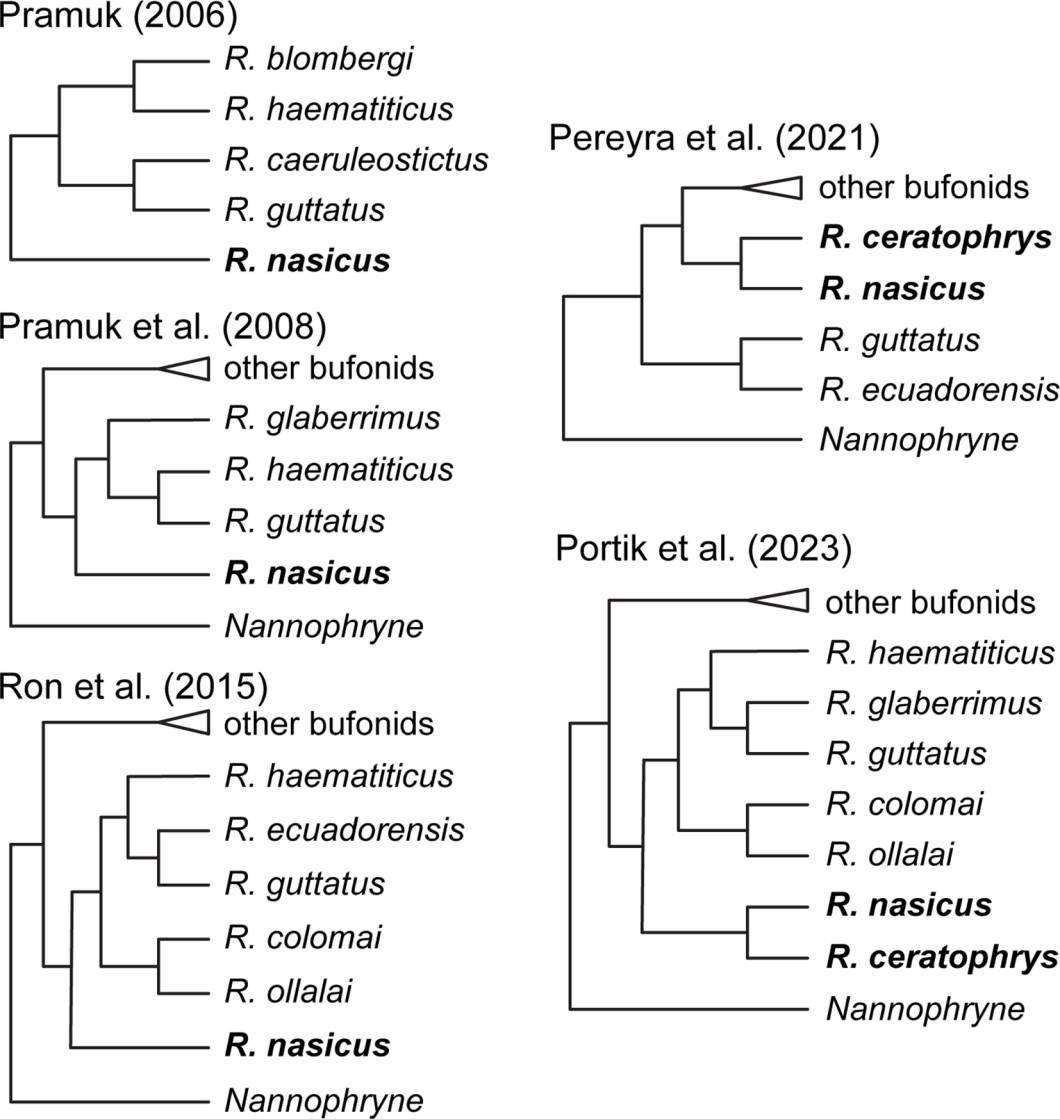
Summarized relationships of “*Rhaebo” nasicus* (and *R. ceratophrys* when included) according to the several published phylogenetic hypotheses for Bufonidae: Pramuk ([121]: Fig. 1; morphological data alone, MP tree); Pramuk et al. ([133]: Fig. 1; molecular data alone, Bayesian analysis tree); Ron et al. ([95]: Fig. 1; molecular data alone, ML tree); Pereyra et al. ([96]: Fig. 10; phenotypic + molecular data, MP tree), and Portik et al. ([97]: Fig. 57; molecular data alone, ML tree)

Ron et al. [95] studied the poorly known genus *Andinophryne* (now included in *Rhaebo*). They performed separate phylogenetic analyses for mitochondrial and nuclear data, placing *Andinophryne olallai* and *A. colomai* within *Rhaebo* —*Rhaebo nasicus* was sister to all other *Rhaebo* + *Andinophryne*. *Rhinella ceratophrys* was not included in that study. According to the authors, they preferred to synonymize *Andinophryne* with *Rhaebo* rather than erecting a new genus for *Rhaebo nasicus*, as their study did not include all *Rhaebo*. Other large-scale studies (e.g., [129–130]) have also recovered *Rhaebo nasicus* as sister to all other *Rhaebo* (Fig. 12).

Pereyra et al. [96] studied the evolution and systematics of *Rhinella* with a large and dense taxon sampling (including an extensive outgroup sampling). They included representatives of *Rhinella ceratophrys* in a phylogenetic analysis for the first time. In their total evidence analysis under Maximum Parsimony ([96]: Fig. 10), they recovered *Rhaebo nasicus* as sister to *Rhinella ceratophrys* and the rest of *Rhaebo* as sister to *Rhaebo nasicus* and “*Rhinella” ceratophrys* + other bufonids, rendering both *Rhinella* and *Rhaebo* non-monophyletic. The authors transferred *R. ceratophrys* to *Rhaebo*, despite the paraphyly of *Rhaebo*, arguing that their analysis was not designed to rigorously test the monophyly of *Rhaebo*.

Recently, Portik et al. [97] published a large study on the phylogeny of anurans. They included seven of the 14 valid species assigned to *Rhaebo*. In their topology, *Rhaebo nasicus* and *Rhaebo ceratophrys* are sister taxa and together form the sister group to all other *Rhaebo*.

In summary, neither *Rhaebo nasicus* or *R. ceratophrys* have ever been recovered as nested within other *Rhaebo* species in any phylogenetic hypothesis (Fig. 12) and the most inclusive analysis of Bufonidae strongly supports the clade formed by *Rhaebo nasicus* and *R. ceratophrys* as the sister clade to all other *Rhaebo*. The larval morphology of other *Rhaebo* species is a generalized, benthic type (e.g., [131–132]), while the larval morphology of *R. nasicus* (and likely *R. ceratophrys*) is a specialized torrential form (see above). Therefore, combining larval and adult morphological synapomorphies for the clade of *R. nasicus* and *R. ceratophrys* (e.g., [96, 119, 120]; this study), along with phylogenetic evidence supporting their monophyly ([96, 97]; this study), we propose that this clade should be recognized as new genus, which is named hereafter.

### Taxonomic account

#### *Adhaerobufo *gen. nov.

##### ZooBank registration

urn: lsid: zoobank.org: act: C757A1FA-A343-4134-8371-6A42797F162A

##### Type species

*Bufo nasicus* (Werner, 1903 [125]) comb. nov.

##### Immediately more inclusive taxon

Bufonidae Gray, 1825 [134].

##### Content

*Adhaerobufo ceratophrys* (Boulenger, 1882 [122]) comb. nov, and *Adhaerobufo nasicus* (Werner, 1903 [125]) comb. nov.

##### Etymology

*Adhaerobufo ***gen. nov.** (gender masculine) is derived from the Latin *adhaerens*, meaning adherent and the Latin *būfo*, meaning toad. The name refers to the unique suctorial morphology of their tadpoles.

##### Definition and diagnosis:

*Adhaerobufo ***gen. nov.** can be differentiated from all other Bufonidae by the combination of the following characters: (1) tadpole with enlarged, suctorial, oral disc; (2) tadpole oral disc with a complete row of marginal papillae; (3) tadpole oral disc with multiple rows of submarginal papillae on the lower lip and by a single row of marginal papillae on the upper lip; (4) tadpole oral disc with an uninterrupted second anterior row of keratodonts; (5) presence of the m. interhyoideus posterior at larval stage; (6) presence of the m. rectus abdominis anterior at larval stage; (7) presence of narial vacuities in the buccopharyngeal cavity at larval stage; (8) projecting, enlarged eyelid in adults; (9) presence of an infraocular cream spot in adults, (10) sphenethmoid relatively narrow, overlapping only the medial ends of the palatines; and (11) posterior process of the prootic prominent and notched.

##### Comment

The genetic diversity observed in *Adhaerobufo ***gen. nov.** strongly suggests the occurrence of at least one additional species within the genus (see Appendix MS3). Given the imprecise type locality of *A. nasicus* and the high genetic divergence observed between the sequences of the tadpole and adult specimens from several different localities, there is likely a hidden diversity in the genus, with more species to be described.

### Further comparisons with other genera

#### Larval characters

*Adhaerobufo ***gen. nov.** presents several of the bufonid larval synapomorphies, such as the absence of the m. diaphragmatopraecordialis, the lateral fibers of m. subarcualis rectus II-IV invading branchial septum IV, the larval lungs being rudimentary or absent, and presence of single pairs of infralabial papillae. Nevertheless, it lacks other bufonid synapomorphies, such as the oral disc with a wide ventral gap in marginal papillae and the absence of the m. interhyoideus posterior.

The complete row of marginal papillae differentiates *Adhaerobufo ***gen. nov.** from all other bufonids, including other members of *Rhaebo*. The enlarged, suctorial disc differentiates *Adhaerobufo ***gen. nov.** from all other bufonids except *Ansonia*, *Blaira*, *Phrynoidis*, and *Werneria*. The lack of a belly sucker differentiates it from *Adenomus*, *Atelopus*, *Bufo* (part; *Bufo aspinus*), *Rhinella* (part; *Rhinella veraguensis* group), and *Sabahphrynus*. The uninterrupted second anterior row of keratodonts differentiates *Adhaerobufo ***gen. nov.** from most genera, except *Amazophrynella*, *Phrynoidis*, and *Werneria* (although some few species within some genera, such as, *Adenomus*, *Ansonia*, *Atelopus*, *Bufo*, *Bufotes*, *Capensibufo*, *Ingerophrynus*, *Melanophryniscus*, *Rhinella*, and *Sclerophrys*, have been reported lacking the interruption). The multiple rows of submarginal papillae in the lower lip and a single row of marginal papillae in the upper lip differentiate *Adhaerobufo ***gen. nov.** from all genera but *Werneria*. The presence of the m. interhyoideus posterior differentiates it from all other bufonids except *Amazophrynella*. Finally, the presence of narial vacuities in the buccopharyngeal cavity differentiates *Adhaerobufo ***gen. nov.** from all other bufonids except *Ansonia*, *Atelopus*, *Incilius* (part; *Incilius coniferus*), *Schismaderma*, and *Werneria*.

Several *Rhaebo* species have their tadpoles described, including *R. glaberrimus*, *R. guttatus*, *R. haematiticus*, and *R. caeruleostictus* (e.g., [131, 132, 135]). None of these species has a suctorial form, and most typify the benthic, lentic type common across bufonids. Several other members of the genus have no published data on tadpole morphology, and to our knowledge no collections have been made. Previous attempts to collect the tadpole of *Rhaebo olallai* have been unsuccessful, and while recently metamorphized froglets were found alongside a fast-flowing mountain stream in the Ecuadorian Andes, no tadpoles were found within the stream ( [136], Trageser S., pers comm).

Finally, we would like to stress that a phenotypically similar tadpole from Amazonia, which shares all external morphology characters with *A. nasicus*, including the color pattern, the enlarged oral disc, and the complete row of marginal papillae, is awaiting formal description (T. Grant and T. Pezzuti, pers comm). Individuals of that species in late Gosner developmental stage [111] present a dorsal color pattern very similar to that of adults of *A. ceratophrys* (thus contra juveniles of *Rhaebo* [95]). Likewise, juveniles of *A. nasicus* have the same color pattern as adults, both in life and in preservative.

#### Adult characters

As noted by Hoogmoed [120], *Adhaerobufo nasicus* and *A. ceratophrys* share a projecting flap above the eyelid. This character is especially pronounced in *A. ceratophrys*, where it is enlarged to form a spiny projection above the eye. The lateral surfaces of head and body (including the ventral portion of the parotoid macroglands) in *Adhaerobufo ***gen. nov.** are dark, similar to some species of *Rhaebo* (e.g., *R. blombergi*, *R. guttatus*, *R. haematiticus*). Nevertheless, both species have a well-defined infraocular cream spot. The combination of dark pattern contrasting with an infraocular cream spot is a putative synapomorphy of *Adhaerobufo ***gen.nov.**, as it does not occur in other related genera of Bufonidae. *Adhaerobufo ***gen. nov.** shares several characters previously associated with *Rhaebo*, including an elongate transverse process of vertebra VI, well-developed omosternum, and large and notched posterior processes of the prootic ([95]: Fig. 6 for *R. blombergi* and *R. colomai*, the authors pers. obs.). *Adhaerobufo ***gen. nov.** differs from *Rhaebo* in having a distinctly narrow sphenethmoid.

### Distribution (Fig. 13)

Northwestern Guyana and eastern Venezuela (*Adhaerobufo nasicus*) and upper Amazon Basin in western Brazil, southeastern Colombia, eastern Ecuador, northeastern Peru, and southern Venezuela (*A. ceratophrys*).

**Fig. 13:**
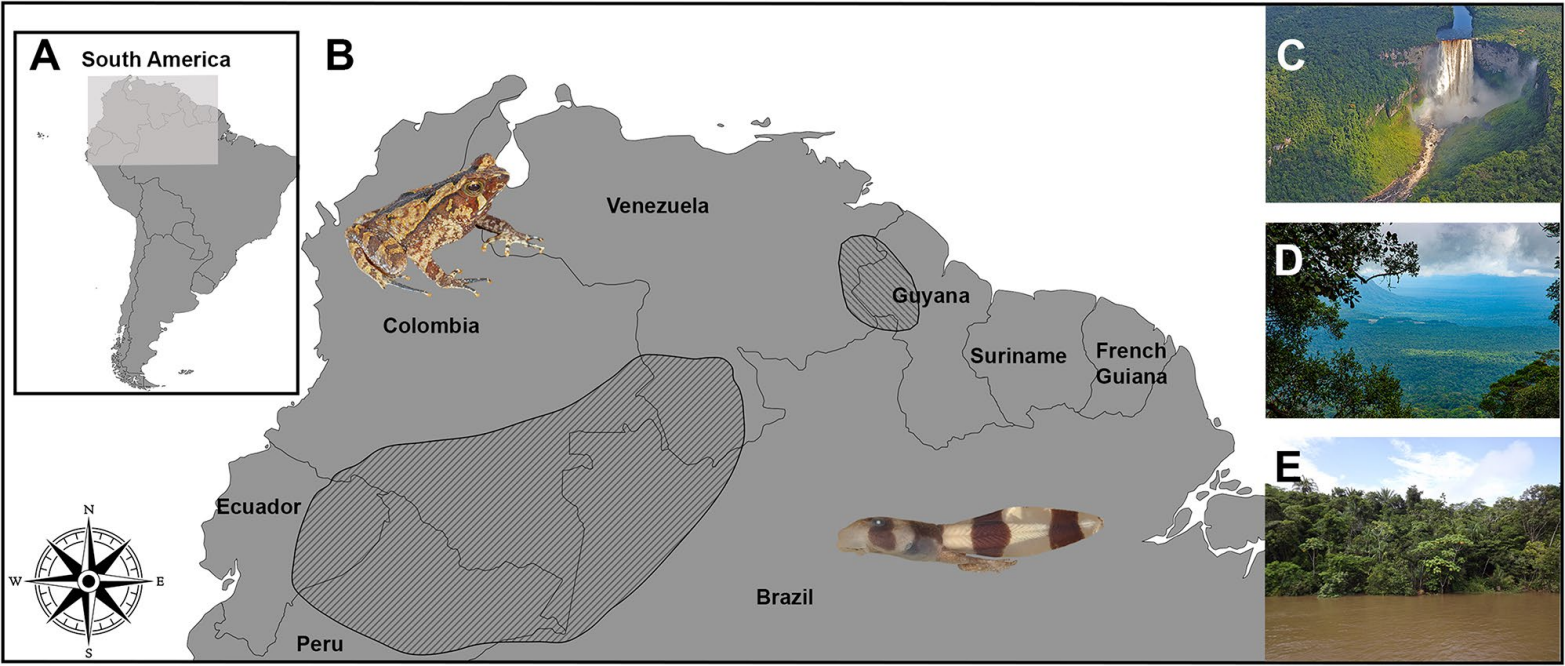
Geographical distribution of *Adhaerobufo ***gen. nov.** in northwestern Guyana, eastern Venezuela and upper Amazon Basin. Inset map of South America, highlighting the geographical area occupied by the genus (**A**). Known distribution of *A. ceratophrys* and *A. nasicus* (**B**). Examples of macrohabitats in which the new genus is present; Kaieteur Falls in Guyana (**C**), uplands and highlands of western Guyana (**D**), and lowlands, Amazon Forest, Icá River, Brazil (**E**). Shape files of the geographical distribution were downloaded from the IUCN website. Adult and tadpole are from *A. nasicus*. Photos by: Philippe Kok (**C** and **D**) and Pedro H. Dias (**E**)

### Natural history

**Fig. 14:**
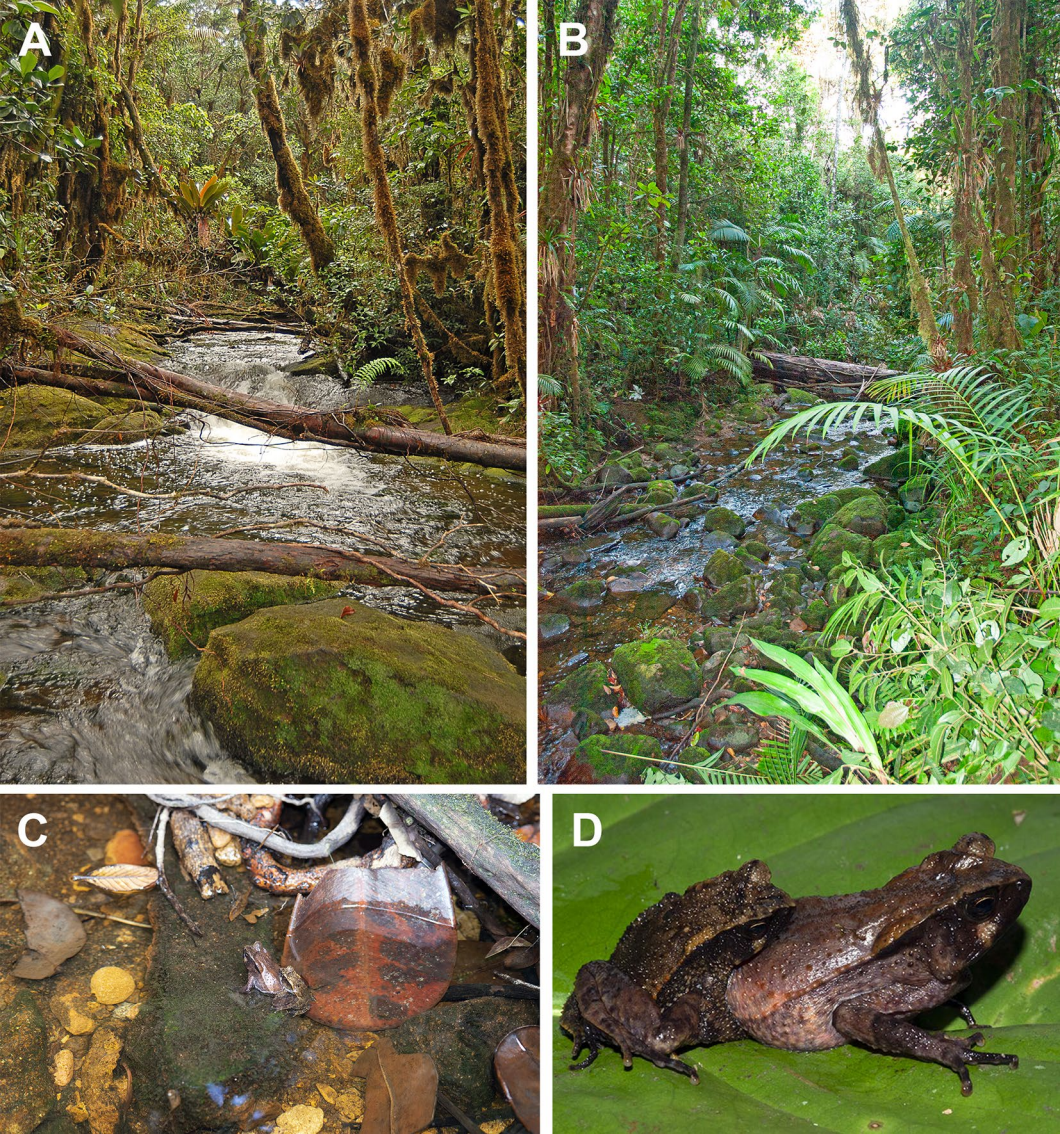
Kamana Creek, upstream within 100 m of Kamana Waterfall, draining Mt. Kopinang low waters where tadpoles of *Adhaerobufo* were collected (**A**) and an unnamed stream on the slopes of Maringma-tepui where tadpoles were also observed (**B**). Amplexing couple of *A. nasicus* (**C** and **D**). Photos by D. Bruce Means (**A, C, D**) and Philippe J. R. Kok (**B**)

Tadpoles of *Adhaerobufo nasicus* were scraped by aquarium-mesh dip-net from the sides of large, submerged boulders of Roraima Supergroup sandstones in the bed of Kamana Creek, upstream within 100 m of Kamana Waterfall (Fig. 14A), draining Mt. Kopinang, one of the peaks of the Wokomung Massif, Potaro-Siparuni District, Guyana. Tadpoles were observed clinging by their mouthparts to the vertical sides of big boulders on 7 December 2006 (DBM-3372); 18 July 2007 (observed when water was shallow on 18 July but not collected on 19 July due to torrential flow overnight); and 25 June 2012 (CPI10704). Figure 12B is a view of an unnamed stream on the slopes of Maringma-tepui on 22 November 2007 where *Adhaerobufo* tadpoles were also observed (same ecological data as above). Figure 14C–D are of an amplexing pair of *A. nasicus* in situ (Wokomung Massif) when first discovered on 20 July 2012 (14 C), and shortly thereafter when placed on a leaf for photography (14D). Amplexus is inguinal, and couples have been observed in shallow waters, on the side of rivers. Tadpoles and adults were observed in similar microhabitats at the base and on the slopes of Maringma-tepui in western Guyana in November 2007 (e.g., Fig. 14B), and in the La Escalera region of Venezuela in November 2010. Adult individuals were collected/observed all year long in Kaieteur National Park (west-central Guyana), although tadpoles were not found at that location. In Kaieteur National Park adults were often found relatively far away from any fast-flowing streams suggesting either periodical migration to suitable breeding sites, or plasticity in egg deposition site. Since we never collected any *A. nasicus* tadpole in non-flowing waterbodies, we favor the first hypothesis.

## Discussion

### Larval morphology, systematics, and taxonomy

The impact of larval morphology on the systematics of bufonids has been widely discussed recently [33, 63]. Larval synapomorphies of Bufonidae are: (1) oral disc with wide ventral gap in marginal papillae; (2) anterolateral process of crista parotica absent; (3) m. diaphragmatopraecordialis absent; (4) lateral fibers of m. subarcualis rectus II–IV invading branchial septum IV; (5) larval lungs rudimentary or absent; (6) the m. interhyoideus posterior absent; and (7) a single pair of infralabial papillae [21, 33, 47, 63]. Additionally, several synapomorphies have been reported for less inclusive clades (e.g., [33, 60, 63, 137, 138]).

*Adhaerobufo nasicus* shares several of these synapomorphies, but reverted some states; for instance, it is characterized by the complete row of marginal papillae and by presenting the m. interhyoideus superior. Additionally, other autapomorphic traits are present in the larvae of *Adhaerobufo ***gen. nov.**, such as (1) the enlarged, suctorial, oral disc; (2) multiple rows of submarginal papillae in the lower lip and by a single row of marginal papillae in the upper lip; and (3) the presence of narial vacuities in the buccopharyngeal cavity. The combination of traits supports *Adhaerobufo ***gen. nov.** in Bufonidae but also distinguishes it from all other bufonids.

**Fig. 15:**
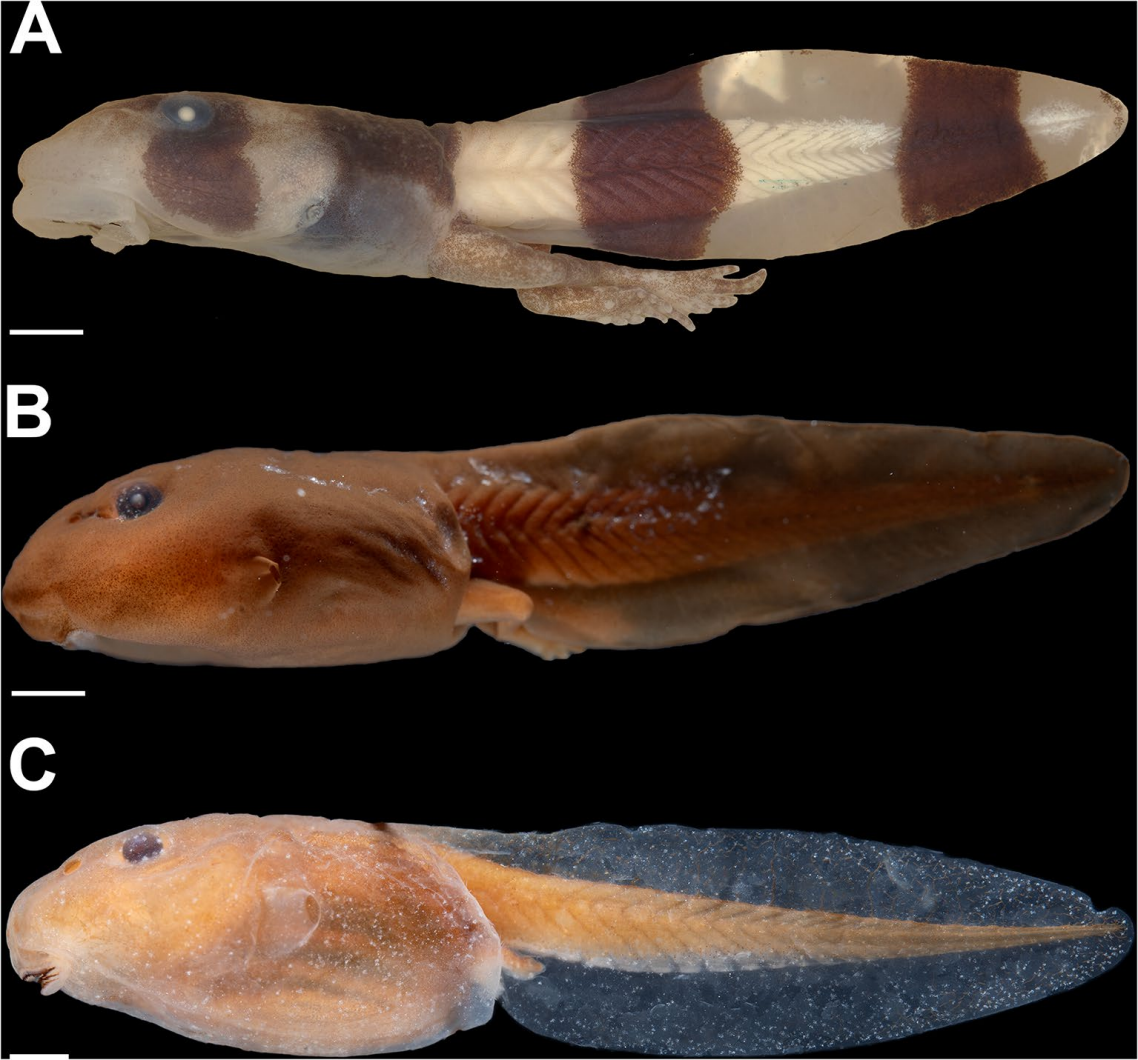
Phenotypic differences between *Adhaerobufo nasicus* (CPI10704) (**A**) and *Rhaebo* larvae; *R. caeruleostictus* (KU112307) (**B**) and *R. haematiticus* (KU68327) (**C**). Note the striking differences in body shape, mouthparts, and coloration. Scale bars = 1.0 mm. Photos by: Pedro H. Dias (**A**) and Jackson Phillips (**B** and **C**)

*Adhaerobufo ***gen. nov.** has been consistently recovered as either sister taxon of *Rhaebo* (e.g., [96, 97, 121].,; this work, Fig. 2) or closely allied to this genus ( [96]; this work Fig. Figure 1) and the morphology of their larvae is unique—especially in comparison with “typical” *Rhaebo* larvae (Fig. 15)—including several apomorphic transformations, supporting our proposition of a new genus. Furthermore, additional characters from adult morphology and osteology also underscore the distinctiveness of this taxon. The genus *Rhaebo* has relatively few potential synapomorphies, and the widened shape of the sphenethmoid has been used previously as an important generic trait. We find that *Adhaerobufo ***gen. nov.** lacks this character, therefore, the inclusion of *A. nasicus* and *A. ceratophrys* in *Rhaebo* would potentially destabilize its taxonomy. Additionally, by recognizing *Adhaerobufo ***gen. nov. **as a new genus, *Andinophryne* can be revalidated without affecting the monophyly of *Rhaebo*. We refrained from making this change, as we have not personally examined specimens of *R. olallai* or *R. colomai*. Furthermore, Ron et al. [95] suggested some phenotypic characters, including a widened sphenethmoid, to support *Andinophryne* as part of *Rhaebo*.

Ron et al. [95] also mentioned that the coloration pattern of juveniles, described as “dorsal coloration consisting of a dark background with contrasting thin clear stripes or dots”, could be a synapomorphy of *Rhaebo*. The fact that (1) tadpoles closely related to *A. ceratophrys* (see above) in late developmental stage already present the adult dorsal color pattern; and (2) that juveniles of *A. nasicus* have the same color pattern as the adults suggests that the synapomorphy proposed by Ron et al. [95] supports the monophyly of *Rhaebo*, including *Andinophryne*. Nevertheless, just as in *Rhaebo* sl, *Peltophryne* juveniles change their color pattern (e.g., [139, 140], which could affect the optimization of that character. Thus, we recommend caution when considering this potential synapomorphy. The present results reinforce the potential of larval morphology in the fields of systematics, taxonomy, and evolution. Tadpoles are highly variable regarding their morphology (e.g., 25, 27, 28, 35, 43, 138, 141, 142), ecology and behavior (e.g., [143, 144]), among others. Such variation makes tadpoles a powerful source of evidence to test hypotheses of evolutionary relationships among frogs. Recently, several studies have approached larval morphology in a phylogenetic context (e.g., [35, 45]), resulting in the identification of novel synapomorphies and strengthening the support of clades.

It is also evident that the exploration of larval morphology in previously unstudied groups has widened our perception of larval diversity. In the past 20 years, astonishing novel phenotypes have been reported (e.g., [27, 28, 141]), but many of these new characters have never been included in any phylogenetic analysis. We strongly advocate for the usage of larval morphology in further studies about the evolution and diversification of anurans.

Finally, we believe that the taxonomy of anurans (and of other organisms with complex life cycles) could greatly benefit from the usage of non-adult semaphoronts. Historically, anuran taxonomists have concentrated their efforts in metamorphosed adult (mainly males), ignoring larval individuals. When such dogma is broken, taxonomists can better delimit, recognize, and describe species, and other supraspecific clades. For example, Grosjean et al. [24] were able to describe *Clinotarsus penelope* (Ranidae), referring a tadpole as the holotype. Our study follows the same path, and larval characters were pivotal for the proposition of *Adhaerobufo ***gen. nov.** – named after larval characteristics.

### The evolution of suctoriality in bufonid tadpoles

Recently, Dias and Anganoy-Criollo [33] discussed the convergent evolution of suctorial and gastromyzophorous ecomorphologies across anurans. They stressed that the presence of enlarged oral disc and/or of a belly sucker were different strategies shaped by natural selection in tadpoles occupying fast-flowing waters. These strategies have evolved independently multiple times across 13 families of anurans. These authors, however, also discussed the differences among these larvae, suggesting that the real diversity of suctorial forms is unknown.

**Fig. 16:**
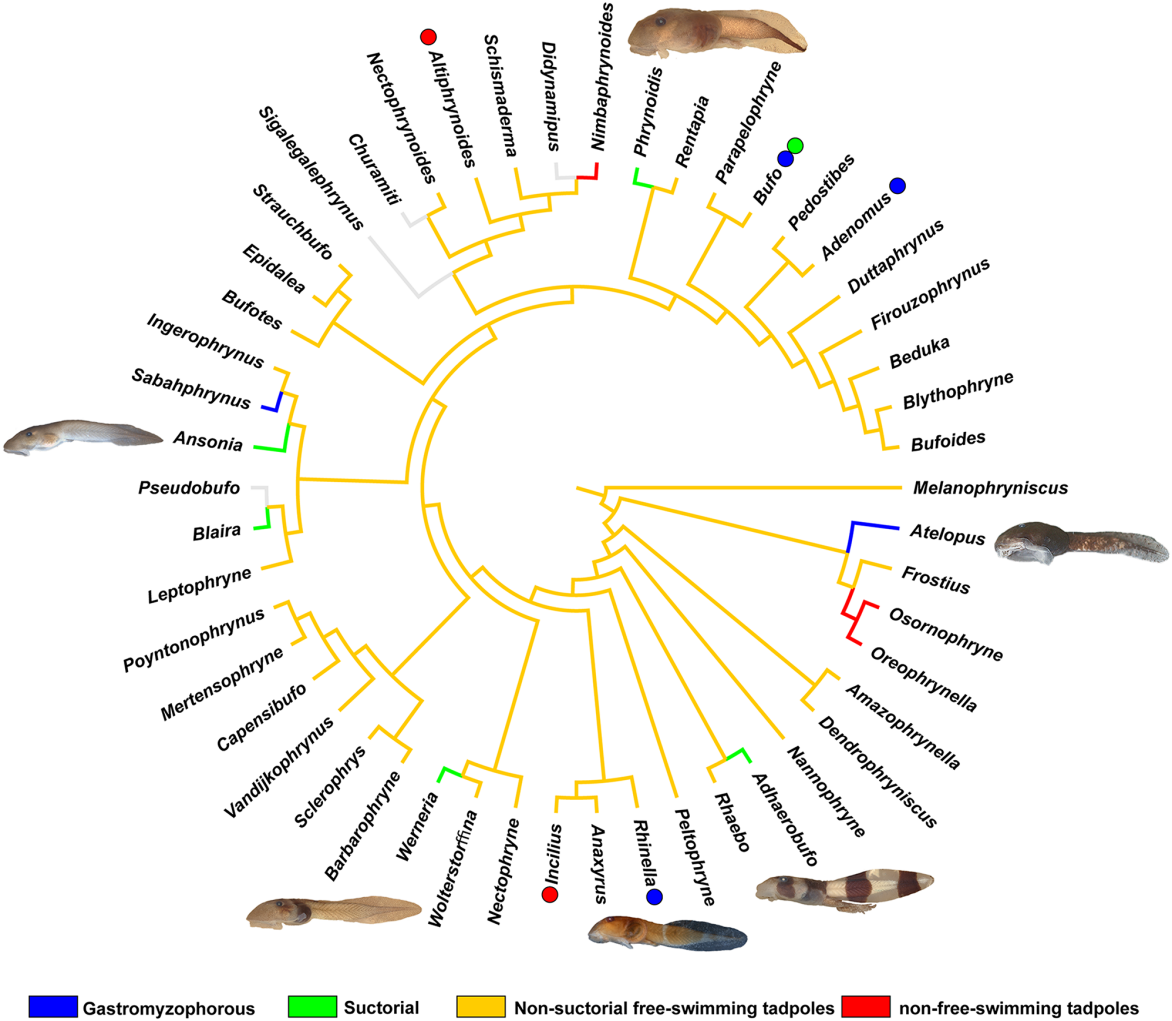
Gastromyzophorous and suctorial larvae evolved independently at least 10 times within bufonids, revealed by the phylogenetic hypothesis of Portik et al. [95] showing the genera in which these tadpoles have evolved. The dots next to the genera indicate derived conditions within them. Photos by: Pedro H. Dias and Jackson Phillips

Suctorial and gastromyzophorous larvae evolved independently at least 10 times in Bufonidae (Fig. 16). Gastromyzophorous tadpoles have been reported in all known *Atelopus* larvae, in three species of the *Rhinella veraguensis* group (*R. chrysophora*, *R. quechua*, and *R. veraguensis*), in *Sabahphrynus maculatus*, in *Adenomus kandianus*, and in *Bufo aspinius* [33, 60, 145–147], whereas suctorial tadpoles have evolved in *Adhaerobufo ***gen. nov.**, *Ansonia*, *Blaira*, *Phrynoidis*, *Bufo pageoti*, *Bufo torrenticola*, *Bufo tuberospinus*, and *Werneria* ([66, 148–151]; the present study).

As stressed by Dias and Anganoy-Criollo [33], suctorial larvae of bufonids share many traits, but also differ widely. Suctorial larvae share a series of convergent traits, such as the presence of a developed element in the prenarial arena and of narial vacuities [33], a widening of the palatoquadrate, enlarged and short cornua trabeculae, robust lower jaw, upper jaw with fused elements and with a well-developed processus posterior dorsalis, adrostral elements often present, reduction of elements of the branchial basket, modifications in the insertion of the abdominal muscles, presence of a rectus abdominis superior, suspensorio-angularis with a sub- or postorbital origin, and well developed axial muscles ( [21, 47, 60, 152]; PHD, the authors, pers. obs.). Each independent instance of bufonid suctoriality is also unique. The most obvious difference among many is the presence of a belly sucker in gastromyzophorous species, as opposed to an enlarged oral disc, but there are other variable states. For instance, jaw sheaths are interrupted in *Ansonia*, but continuous in other taxa. Other variable characters are the presence and distribution of submarginal papillae, tail tip morphology, and body color pattern. Despite the great potential of this system in the study of novelty and ecomorphological evolution, the significance of such variation remains largely unexplored.

**Fig. 17:**
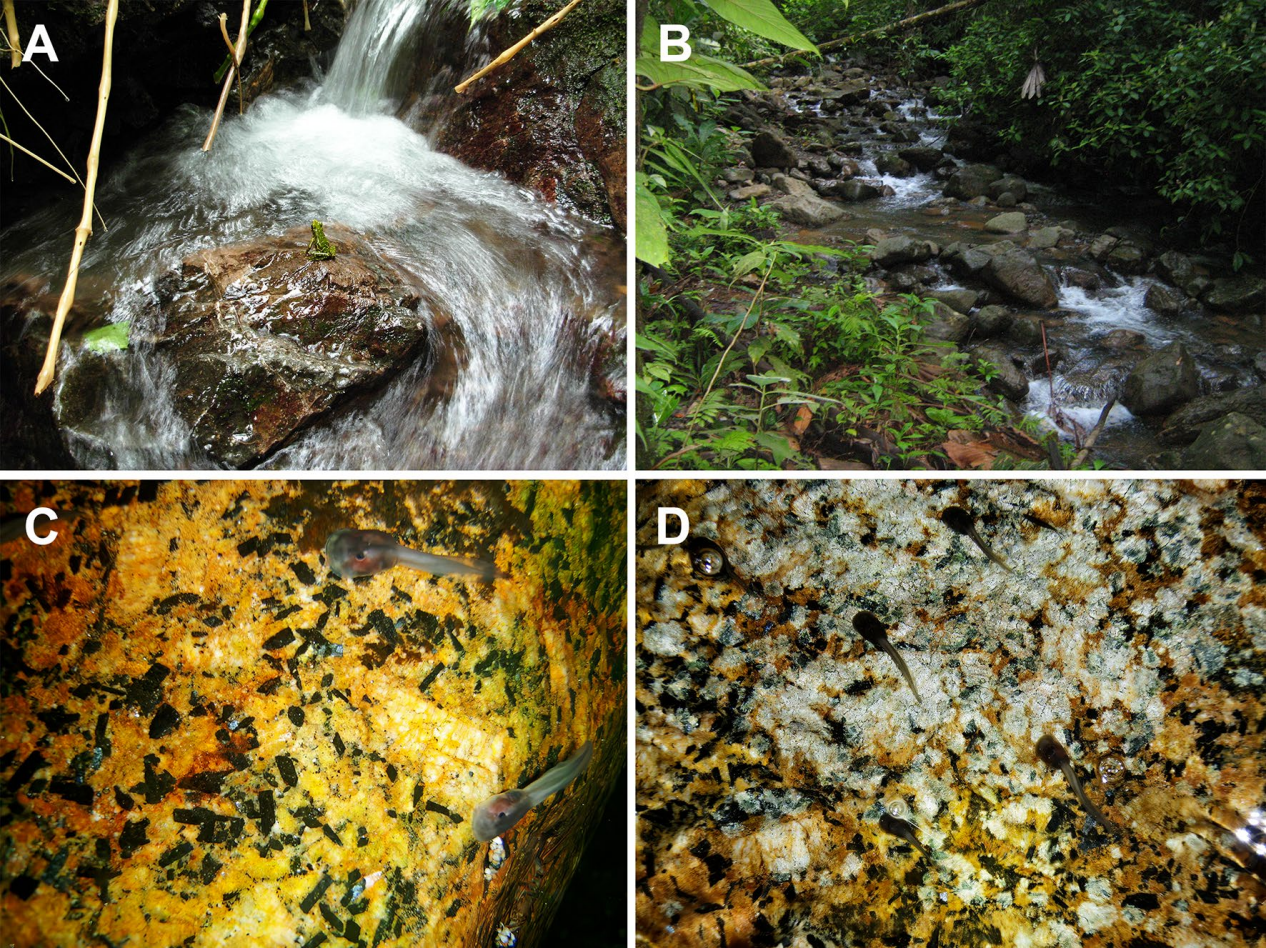
Torrential environments that were colonized by suctorial/gastromyzophorous larvae of bufonids. Adult of *Atelopus* sp. in Tacarcuna, Colombia (**A**); fast flowing waters occupied by *Atelopus elegans* at Isla Gorgona, Colombia (**B**); larvae of *Ansonia guibei* attached to rocks of fast flowing streams in Borneo (**C** and **D**). Photos by Marco A. Rada (**A**), David Velázquez (**B**), and Alexander Haas (**C** and **D**)

*Adhaerobufo***gen. nov.** represents an interesting case, given that the adult form is rather unspectacular, being confused with many unrelated bufonid sub-clades over time. It is remarkable that the adult form appears to be so unaffected by radical evolutionary changes to the larva. We interpret this as further evidence of the decoupling power of metamorphosis, whereby evolutionary changes in the larval form can operate semi-independently of the adult phenotype, despite sharing a genome and being part of the same developmental sequence [153–154]. From a macroevolutionary perspective, it is interesting to note that the evolution of suctoriality may be particularly common in bufonids. Vera Candioti et al. [57] demonstrated that suctorial forms are the exclusive larval form of four anuran families, (Ascaphidae, Conrauidae, Heleophrynidae, and Nasikabatrachidae), and that while suctoriality has evolved in several other families, it is a relatively rare phenomenon in anurans (number of suctorial species/number of species). Future studies that identify the features that make suctoriality a more common evolutionary outcome in some lineages (including bufonids) than others could provide insight into not only adaptive ecomorphological evolution, but also non-adaptive factors that limit such evolutionary changes.

## Conclusion

We describe the tadpole of “*Rhaebo*”* nasicus* and present evidence supporting the erection of a new genus, *Adhaerobufo ***gen. nov.**, to recognize the evolutionary distinctiveness of this group of South American toads. The tadpole of *Adhaerobufo nasicus* is a brightly colored, suctorial form adapted to living in fast-flowing streams. The oral morphology of that tadpole is unique among bufonids, with a complete row of marginal papillae that differentiates it from all other tadpoles known from the family Bufonidae. Suctorial larvae have evolved independently at least 10 times in bufonids; in each case, a combination of convergent and unique traits can be observed. Our findings echo the importance of tadpoles in systematic and taxonomic studies.

## Electronic supplementary material

Below is the link to the electronic supplementary material.

Supplementary Material 1: Appendix MS1 take ESM 1

Supplementary Material 2: Appendix MS2 take ESM 2

Supplementary Material 3: Appendix MS3 take ESM 3

Supplementary Material 4: Appendix MS4 take ESM 4

Supplementary Material 5: Figure MS5 take ESM 5

Supplementary Material 6: Figure MS6 take ESM 6

## Data Availability

Further information and requests for additional resources should be directed to and will be fulfilled by the corresponding authors. This article has been registered in ZooBank with the life science identifier urn: lsid: zoobank.org: pub: 95161DF6-0C46-4CC1-9BD1-045545B57C57.

## Abbreviations

ALPH: Antelateral process hyalis
AP: Articular process
APH: Anterior process hyalis
AT: Adrostral tissue
BFA: Buccal floor arena
BFAP: Buccal floor arena papillae
BRAP: Buccal roof arena papillae
CB: Constrictor branchialis
CH: Ceratohyal
DV: Dorsal velum
HA: Hyoangularis
HP: Hypobranchial plate
HQP: Hyoquadrate process
IH: Interhyoideus
IHP: Interhyoideus posterior
ILP: Infralabial papillae
IM: Intermandibularis
IN: Internal nares
IR: Infrarostral cartilage
JAF: Jackknife absolute frequencies
JF: Jugular foramen
JGC: Parsimony jackknife frequency differences
LMA: Levator mandibulae articularis
LMEP: Levator mandibulae externus profundus
LMES: Levator mandibulae externus superficialis
LMI: Levator mandibulae internus
LMLS: Levator mandibulae longus superficialis
LP: Lateral process
LR: Lateral ridge
LTRF: Labial tooth row formula
MC: Meckel’s cartilage
µCT: High-Resolution micro-computed tomography
ML: Maximum likelihood
MP (in larval cranium): Maximum parsimony
MP (in phylogenetics): Muscular process
MR: Median ridge
NV: Narial vacuities
OH: Orbitohyoideus
OC: Otic capsule
PCM: Proximal commissure
PNP: Postnarial arena papillae
PP: Posterior process
PU: Process urobranchialis
QA: Quadrato-angularis
QOC: Quadro-Orbital commissure
RA: Rectus abdominis
RAA: Rectus abdominis anterior
RC: Rectus cervicis
SA: Suprarostral ala
SAR I: Subarcualis rectus I
SAR II–IV: Subarcualis rectus II–IV
SB: Subocular bar
SC: Suprarostral copora
SH: Suspensoriohyoideus
SO: Subarcualis obliquus
SP: Spicule
TA: Tongue anlage
TH: Trabecular horns
TP: Triangular projection
TS: Tectum synoticum
TTM: Taenia tecti medialis
TTT: Taenia tecti transversalis
UFBoot: Ultrafast Bootstrap Approximation
UJ: Upper Jaw Sheath
VV: Ventral Velum
