# Supplementary figures and images for: The remarkable larval morphology of Rhaebo nasicus (Werner, 1903) (Amphibia: Anura: Bufonidae) with the erection of a new bufonid genus and insights into the evolution of suctorial tadpoles
Source: Zoological Lett. 2024 Sep 30;10:17. doi: 10.1186/s40851-024-00241-0 (PMC11440901; doi:10.1186/s40851-024-00241-0)

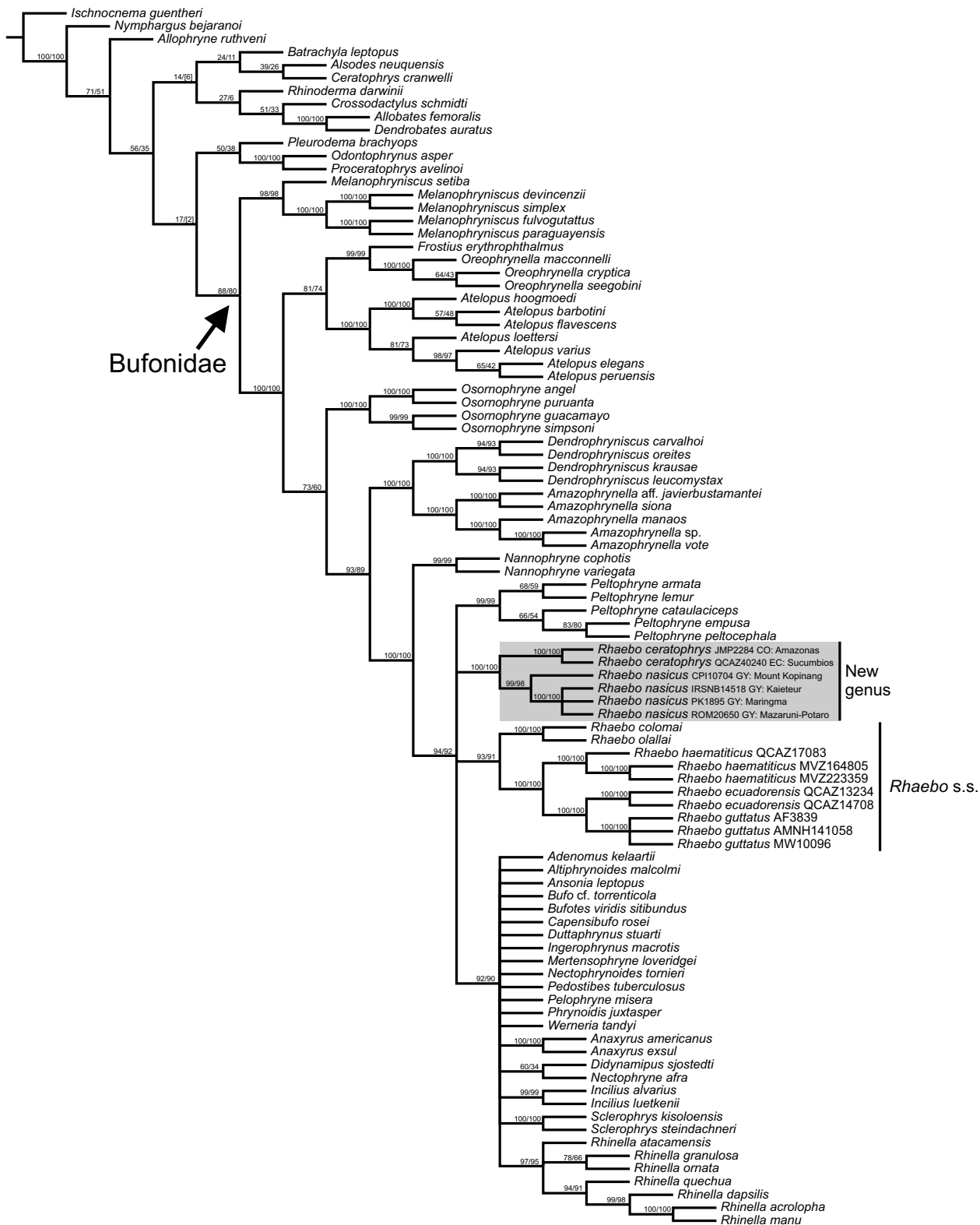

Supplement: Supplementary file 1 — Supplementary Material 1: Appendix MS1 take ESM 1 [file 40851_2024_241_MOESM1_ESM.pdf]

Bufo

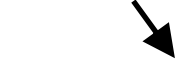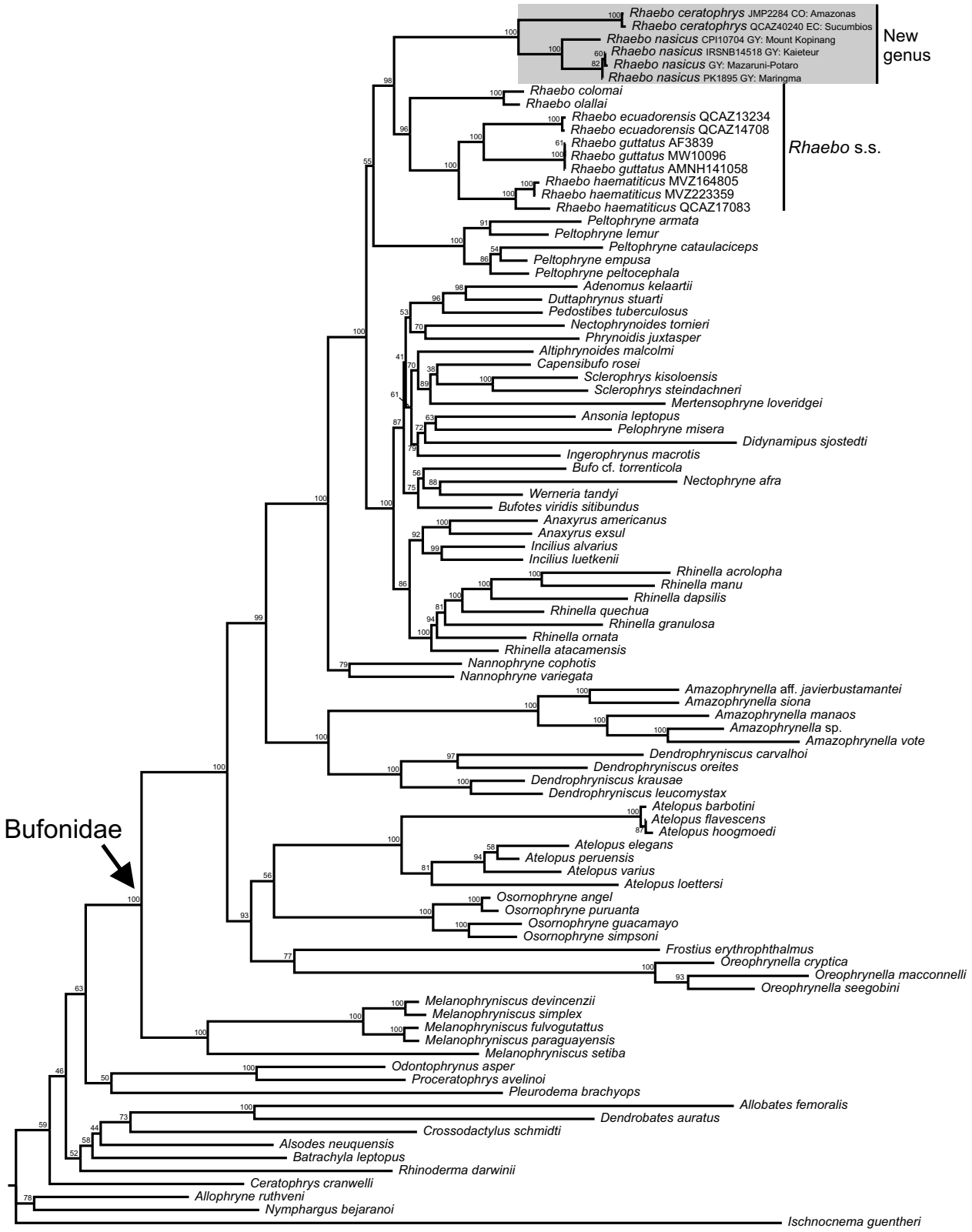

0.04

Supplement: Supplementary file 2 — Supplementary Material 2: Appendix MS2 take ESM 2 [file 40851_2024_241_MOESM2_ESM.pdf]
