# Supplementary material for: The remarkable larval morphology of Rhaebo nasicus (Werner, 1903) (Amphibia: Anura: Bufonidae) with the erection of a new bufonid genus and insights into the evolution of suctorial tadpoles
Source: Zoological Lett. 2024 Sep 30;10:17. doi: 10.1186/s40851-024-00241-0 (PMC11440901; doi:10.1186/s40851-024-00241-0)
Supplement: Supplementary file 4 — Supplementary Material 4: Appendix MS4 take ESM 4 [file 40851_2024_241_MOESM4_ESM.docx]

**Best partition scheme and best-fit models selected by ModelFinder for the molecular data.**

| **Data blocks** | **Model** |
| --- | --- |
| *12s-tRNAval-16s* | GTR+F+I+R5 |
| *cxcr4*_1, *pomc*_2 | HKY+F+R3 |
| *cxcr4*_3, *pomc*_1, *pomc*_3, *rag1*_2, *rag1*_3 | TPM3u+F+I+R2 |
| *rag1*_1 | HKY+F+G4 |
