# Supplementary material for: The remarkable larval morphology of Rhaebo nasicus (Werner, 1903) (Amphibia: Anura: Bufonidae) with the erection of a new bufonid genus and insights into the evolution of suctorial tadpoles
Source: Zoological Lett. 2024 Sep 30;10:17. doi: 10.1186/s40851-024-00241-0 (PMC11440901; doi:10.1186/s40851-024-00241-0)
Supplement: Supplementary file 5 — Supplementary Material 5: Figure MS5 take ESM 5 [file 40851_2024_241_MOESM5_ESM.docx]

**Pairwise distance matrix (uncorrected p-distances) for 16S sequences of *“Rhaebo*”.**

|  | 1 | 2 | 3 | 4 | 5 | 6 | 7 | 8 | 9 | 10 | 11 | 12 | 13 | 14 | 15 |
| --- | --- | --- | --- | --- | --- | --- | --- | --- | --- | --- | --- | --- | --- | --- | --- |
| 1 - *R. ceratophrys* JMP2284 | – |  |  |  |  |  |  |  |  |  |  |  |  |  |  |
| 2 - *R. ceratophrys* QCAZ40240 | 0.2 | – |  |  |  |  |  |  |  |  |  |  |  |  |  |
| 3 - *R. nasicus* IRSNB14518 | 9.0 | 9.0 | – |  |  |  |  |  |  |  |  |  |  |  |  |
| 4 - *R. nasicus* PK1895 | 9.2 | 9.2 | 0.2 | – |  |  |  |  |  |  |  |  |  |  |  |
| 5 - *R. nasicus* ROM20650 | 9.6 | 9.6 | 0.2 | 0.4 | – |  |  |  |  |  |  |  |  |  |  |
| 6 - *R. nasicus* CPI10704 | 8.8 | 8.8 | 5.0 | 4.8 | 5.6 | – |  |  |  |  |  |  |  |  |  |
| 7 - *R. colomai* | 17.5 | 17.5 | 16.2 | 16.5 | 16.0 | 15.5 | – |  |  |  |  |  |  |  |  |
| 8 - *R. olallai* | 13.6 | 13.6 | 13.0 | 13.2 | 13.6 | 12.6 | 4.7 | – |  |  |  |  |  |  |  |
| 9 - *R. ecuadorensis* QCAZ13234 | 13.4 | 13.4 | 11.9 | 12.1 | 12.4 | 12.9 | 11.5 | 9.4 | – |  |  |  |  |  |  |
| 10 - *R. ecuadorensis* QCAZ14708 | 13.2 | 13.2 | 11.7 | 11.9 | 12.2 | 12.7 | 11.2 | 9.2 | 0.2 | – |  |  |  |  |  |
| 11 - *R. guttatus* AMNH141058 | 14.3 | 14.3 | 12.5 | 12.7 | 13.1 | 13.7 | 11.8 | 10.4 | 6.6 | 6.4 | – |  |  |  |  |
| 12 - *R. guttatus* MW10096 | 14.3 | 14.3 | 12.5 | 12.7 | 13.1 | 13.7 | 11.8 | 10.4 | 6.6 | 6.4 | 0.0 | – |  |  |  |
| 13 - *R. haematiticus* MVZ164805 | 14.3 | 14.3 | 13.5 | 13.7 | 14.1 | 11.9 | 13.1 | 10.2 | 8.5 | 8.3 | 8.1 | 8.1 | – |  |  |
| 14 - *R. haematiticus* MVZ223359 | 13.9 | 13.9 | 13.1 | 13.3 | 13.7 | 11.5 | 13.1 | 10.0 | 8.2 | 8.0 | 7.7 | 7.7 | 0.4 | – |  |
| 15 - *R. haematiticus* QCAZ17083 | 13.7 | 13.5 | 13.6 | 13.8 | 14.2 | 13.8 | 14.3 | 11.6 | 12.2 | 12.0 | 12.9 | 12.9 | 13.9 | 13.8 | – |
