# Supplementary material for: The remarkable larval morphology of Rhaebo nasicus (Werner, 1903) (Amphibia: Anura: Bufonidae) with the erection of a new bufonid genus and insights into the evolution of suctorial tadpoles
Source: Zoological Lett. 2024 Sep 30;10:17. doi: 10.1186/s40851-024-00241-0 (PMC11440901; doi:10.1186/s40851-024-00241-0)
Supplement: Supplementary file 6 — Supplementary Material 6: Figure MS6 take ESM 6 [file 40851_2024_241_MOESM6_ESM.docx]

**Appendix MS4 - Additional specimens examined**

**Acronyms:** **AMNH**, American Museum of Natural History; **ICN**, Instituto de Ciencias Naturales, Universidad Nacional de Colombia; **IRSNB**, Institut Royal des Sciences Naturelles de Belgique; **KU**, University of Kansas Natural History Museum; **UF**, Florida State Museum (formerly FSM), University of Florida; **USNM**, National Museum of Natural History; **UTA**, University of Texas, Arlington Amphibian and Reptile Diversity Research Center; **ZMB**, Zoologisches Museum Berlin; **ZMH**, Zoological Museum Hamburg.

**Tadpoles**

*Ansonia hanitschi*, ZMHA08803

*Atelopus nahumae*, ICN33202

*Nannophryne variegata*, AMNH81404

*Peltophryne peltocephala*, AMNH38451

*Phrynoidis juxtasper*, ZMH653F

*Rhaebo glaberrimus*, ICN49629

*Werneria mertensiana*, ZMB79695

*Rhaebo caeruleostictus*, KU112307

*Rhaebo haematiticus*, KU68327

*Rhinella chrysophora*, USNM592425

**Adults**

*Adhaerobufo nasicus*, IRSNB14518 (Morphosource ID 665847)

*Amazophrynella manaos*, IRSNB15817 (Morphosource ID M98122)

*Rhaebo blombergi*, UF104602 (Morphosource ID M42667)

*Rhaebo haematiticus*, ICN10781

*Rhaebo haematiticus*, UTA-A57572 (Morphosource ID 000658059)

*Rhaebo haematiticus*, UTA-A57579 (Morphosource ID 000658064)

*Nannophryne variegata*, USNM15124
